# Optimizing the use of adsorbent resin for the amelioration of smoke tainted wine

**DOI:** 10.1016/j.fochx.2026.103772

**Published:** 2026-03-19

**Authors:** Yiming Huo, Renata Ristic, David Wollan, Manuella Cazelato Pires, Lukas Gerstweiler, Richard Muhlack, Markus Herderich, Kerry Wilkinson

**Affiliations:** a Discipline of Wine Science, Adelaide University, PMB 1, Glen Osmond, SA 5064, Australia; b Waite Research Institute, Adelaide University, PMB 1, Glen Osmond, SA 5064, Australia; c VAF Memstar, PO Box 794, Nuriootpa, SA 5355, Australia; d School of Chemical Engineering, Adelaide University, North Tce, Adelaide, SA 5005, Australia; e The Australian Wine Research Institute, PO Box 46, Glenside, SA 5065, Australia

**Keywords:** Adsorbent resin, Phenolic glycoconjugates, Resin regeneration, Sensory analysis, Smoke taint, Volatile phenols

## Abstract

The most effective strategies for amelioration of smoke tainted wine involve removal of smoke taint compounds (i.e., free and glycosylated phenols) by adsorbent materials. Ideally, adsorbents can be re-used if taint compounds are subsequently desorbed via a regeneration process. However, a recent study found phenolic glycosides carried over between remedial treatments that used adsorbent styrene-divinylbenzene resin (packed in a fixed-bed column), despite resin regeneration. This suggested inadequate regeneration, such that residual taint compounds remained on the resin. This study optimized resin regeneration by comparing the desorption of free and glycosylated phenols using different eluents, i.e.: aqueous sodium hydroxide vs aqueous sodium carbonate and aqueous ethanol vs aqueous isopropanol. Free and glycosylated phenols were effectively desorbed (i.e., >99% and 82–100% desorption, respectively, with no evidence of carryover) when resin was regenerated with 5 bed volumes of 2% sodium hydroxide, followed by 3–5 bed volumes of either 20% ethanol or isopropanol. Subsequent elution of regenerated resin with model wine (12% aqueous ethanol, pH 3.4) confirmed there was no further desorption (carryover) of smoke taint compounds. Following optimization of resin regeneration, nanofiltration and resin treatments were applied to three wines with varying levels of smoke taint. Analysis of untreated and treated wines demonstrated the efficacy of both amelioration and resin regeneration, i.e., ∼50% volatile phenol removal was achieved, improving sensory profiles (smoke-related aromas and flavors were less perceptible), with no saturation or carryover of phenolic glycosides. Research findings offer winemakers an improved approach to remediation of smoke tainted wine.

## Highlights

• Smoke tainted wines were treated using membrane filtration and adsorbent resin.
• Despite resin regeneration, glycosylated phenols were carried over between treatments.
• Desorption of free and glycosylated phenols was optimized using different eluents.
• Both alkaline and alcoholic eluents were required to regenerate saturated resin.
• Subsequent nanofiltration and resin treatment remediated wine, without ‘carryover’.

## 1 Introduction

Wine is a popular beverage with a long history of consumption around the world. It is enjoyed for its diverse sensory qualities, but occasionally faults and taints occur, e.g., cork taint, due to the extraction of 2,4,6,-trichloroanisole from contaminated cork closures (Alvarez-Rodriguez et al., 2002), ladybug taint, due to alkyl-methoxypyrazines derived from insects that can be inadvertently harvested with fruit (Pickering & Botezatu, 2021), and smoke taint, due to adsorption of volatile phenols (guaiacols, phenol, cresols, and syringols), from smoke (Hayasaka et al., 2010; Parker et al., 2012; Ristic et al., 2016). Smoke taint occurs when volatile phenols are adsorbed by grapes during vineyard exposure to bushfire smoke. Volatile phenols have been shown to accumulate in grapevine leaves and fruit as glycosides (Hayasaka et al., 2010; Noestheden, Thiessen, Dennis, Tiet, & Zandberg, 2017; Szeto et al., 2020), i.e., with one or more sugar moieties, due to the action of glycosyltransferase enzymes (Hewitt, Aragon, Ashmore, Collins, & Dhingra, 2024). Wines made from smoke-affected grapes typically exhibit diminished fruit expression, and unpleasant smoky, medicinal, and ashy characters (Ristic et al., 2016), which are rejected by consumers, even after considerable dilution/blending (Bilogrevic et al., 2023). As such, smoke taint has led to significant economic losses and due to the warmer, drier conditions associated with climate change, bushfires are predicted to occur more frequently in coming decades (Clarke, Lucas, & Smith, 2013; Luo, Wang, de Jong, & Flannigan, 2024). Winemakers therefore need techniques for managing the impacts of vineyard smoke exposure, and consequently, smoke tainted wine, to offset revenue losses where vineyard exposure to smoke cannot be avoided or prevented.

The addition of adsorbents such as activated carbon remains the most effective method for amelioration of smoke taint (Culbert et al., 2021; Fudge, Schiettecatte, Ristic, Hayasaka, & Wilkinson, 2012). However, the use of non-selective adsorbents inevitably results in the loss of some desirable wine constituents (Culbert et al., 2021; Fudge et al., 2012); color, aroma and flavor compounds, in particular. Strategies that can selectively remove smoke taint compounds (i.e., volatile phenols and their glycoconjugates) are therefore preferable.

The combined use of membrane filtration and solid phase adsorption (Fig. S1) has emerged as a novel approach to the amelioration of smoke tainted wine (Fudge, Ristic, Wollan, & Wilkinson, 2011; Huo et al., 2025). Originally evaluated in 2011 (Fudge et al., 2011), guaiacol and 4-methylguaiacol were shown to permeate a reverse osmosis membrane, whereby they were removed by eluting the resulting permeate through an adsorbent resin. Larger wine constituents, including phenolic compounds and anthocyanins remained in the retentate, and therefore, the final wine after retentate and treated permeate were blended (Fudge et al., 2011). Recent studies have investigated the partitioning of wine constituents using ultrafiltration membranes of different nominal molecular weight cut-off (MWCO) specifications, with larger constituents (e.g., protein, phenolics, polysaccharides and anthocyanins) being retained by the membrane due to a combination of size exclusion and membrane fouling (Angela, Wollan, Muhlack, Bindon, & Wilkinson, 2024; Conidi, Cassano, Caiazzo, & Drioli, 2017; Sui et al., 2022). More recently, partitioning of smoke taint compounds (free and glycosylated phenols) during nanofiltration (NF) and ultrafiltration (UF) was investigated (Huo et al., 2025). Volatile phenols (having molecular masses of < 200 Da) readily permeated both NF and UF membranes, whereas larger phenolic glycosides (with molecular masses ≥ ∼450 Da (Hayasaka et al., 2013)) were progressively concentrated in retentate as membrane MWCO decreased, such that NF-derived retentate comprised most of the phenolic glycoconjugate pool (Huo et al., 2025).

Polymeric resins have been used as ‘broad-spectrum’ adsorbents for the removal of phenol and various other organic pollutants from water (Caetano, Valderrama, Farran, & Cortina, 2009; Liu & Sun, 2021; Víctor-Ortega, Ochando-Pulido, & Martínez-Férez, 2016). The ability to regenerate adsorbent resins, e.g., through solvent washing (Caetano et al., 2009), reduces waste streams and therefore provides an advantage over other sorptive materials, such as activated carbon. The regeneration of resins has been investigated and both the pH (Víctor-Ortega et al., 2016) and proportion of organic solvent (Liu et al., 2021) in regenerants were found to determine the success and extent of regeneration. During recent trials involving the use of adsorbent resin (i.e., styrene-divinylbenzene beads, approved by OIV (Resolution OIV-OENO 657–2023) for remediation of smoke tainted wine), phenolic glycosides were found to carryover from one wine to the next, despite resin regeneration between treatments (Huo et al., 2025). This suggests the protocol currently used for regeneration does not adequately desorb glycosylated phenols, and therefore requires optimization.

This study therefore focused on understanding and optimizing the desorption of both free and glycosylated phenols from the resin used for amelioration of smoke taint. Particular attention was given to eluent composition, i.e., the pH and strength of alkaline eluents, and also the strength of alcoholic eluent. Experimental design also considered commercial implications, (i.e., solvent availability, safety/food grade and flammability). Research findings from this study provide valuable insight into strategies for adsorption and desorption of smoke taint compounds, and therefore advance methods for amelioration of smoke taint.

## 2 Materials and methods

### 2.1 Chemicals

GC- and HPLC-grade solvents were sourced from Sigma Aldrich (Castle Hill, NSW, Australia) or Merck (Darmstadt, Germany). Other analytical-grade chemicals were sourced from Rowe Scientific (Lonsdale, SA, Australia). Food-grade ethanol (95%) was purchased from Tarac Technologies (Nuriootpa, SA, Australia), and food-grade citric acid was sourced from Winequip Australia (Dudley Park, SA, Australia). Deuterium labelled standards (*d*_4_-guaiacol, *d*_3_-syringol, *d*_5_-*o*-cresol, *d*_6_-phenol and *d*_6_-syringol gentiobioside, >95% purity) for gas chromatography–mass spectrometry (GC–MS) and high-performance liquid chromatography tandem mass spectrometry (HPLC-MS/MS), were purchased from LGC Standards (Manchester, NH, USA).

### 2.2 Resin regeneration trials

A series of regeneration trials (Table 1) were performed (in duplicate) to understand and optimize the desorption of free and glycosylated phenols from the adsorbent resin, which was obtained from VAF Memstar (Nuriootpa, SA, Australia).

**Table 1:** Eluent composition for resin regeneration trials.

Trial	Desorption eluent (volume)	Neutralization eluent (volume)	Carryover eluent (volume)
Preliminary trial (BV = 200 mL)	2% sodium hydroxide (5 BVs)	2% citric acid (5 BVs)	model wine (5 BVs)
pH trial (BV = 50 mL)	2% sodium hydroxide (9 BVs)	2% citric acid (3 BVs)	model wine (3 BVs)
	5% sodium carbonate, pH 12 (9 BVs)	2% citric acid (3 BVs)	model wine (3 BVs)
	5% sodium carbonate, pH 10 (9 BVs)	2% citric acid (3 BVs)	model wine (3 BVs)
	5% sodium carbonate, pH 8 (9 BVs)	2% citric acid (3 BVs)	model wine (3 BVs)
Solvent trial (BV = 50 mL)	6% ethanol (12 BVs)	na	model wine (3 BVs)
	12% ethanol (12 BVs)	na	model wine (3 BVs)
	20% ethanol (12 BVs)	na	model wine (3 BVs)
	6% isopropanol (12 BVs)	na	model wine (3 BVs)
	12% isopropanol (12 BVs)	na	model wine (3 BVs)
	20% isopropanol (12 BVs)	na	model wine (3 BVs)
Optimization trial (BV = 50 mL)	2% sodium hydroxide (9 BVs)	2% citric acid in 20% ethanol (6 BVs)	na
	5% sodium hydroxide (9 BVs)	2% citric acid in 20% ethanol (6 BVs)	na

Model wine = 12% aqueous ethanol saturated with KHT and pH adjusted to 3.4. na = not applicable.

In a preliminary trial, the resin used to remediate smoke tainted wine in a previous study (the breakthrough study reported in Huo et al., 2025) was packed in a fixed-bed column (BV = 200 mL, ∼125 g; Hawach Scientific, Xi'an, China) and washed using an existing regeneration protocol (Table 1), being: elution of 2% aqueous sodium hydroxide (5 BVs), followed by 2% aqueous citric acid (5 BVs), after which, model wine (12% aqueous ethanol saturated with potassium bitartrate (KHT) and pH adjusted to 3.4) was eluted (5 BVs) to determine to what extent there was carryover of any smoke taint compounds. A Masterflex peristaltic pump (Cole-Palmer, Vernon Hills, IL, USA) was used to maintained flow rates of 2.5 BV/h, with each BV of effluent (i.e., 200 mL fractions) collected for compositional analysis.

The optimal eluent compositions for desorption of free and glycosylated phenols were subsequently determined. Used resin from the previous remediation trial (as above) was again packed in a fixed-bed column (BV = 50 mL, ∼30 g) and various alkaline and/or alcoholic solutions (Table 1) eluted (at 2.5 BV/h) using the Masterflex peristaltic pump. Again, each BV of effluent was collected for compositional analysis.

In a pH trial, resin regeneration was attempted by eluting 9 BVs of either 2% aqueous sodium hydroxide or 5% aqueous sodium carbonate (pH adjusted to 8, 10 or 12, with citric acid or sodium hydroxide), followed by 2% aqueous citric acid (3 BVs), after which model wine (3 BVs) was again eluted to determine if there was carryover of volatile phenols. In a solvent trial, resin regeneration involved eluting 12 BVs of 6%, 12% or 20% aqueous ethanol or aqueous isopropanol, followed by model wine (3 BVs) to determine if there was carryover of phenolic glycosides.

In a final optimization trial, the efficacy of resin regeneration was compared following elution of 2% vs 5% aqueous sodium hydroxide (9 BVs), and then 2% citric acid in 20% aqueous ethanol (6 BVs), i.e., a combined neutralization and organic washing step (Table 1). After sampling for compositional analysis, acidified aqueous alcohol effluent (i.e., fractions 11 to 15) were pooled and sampled again, before activated carbon (2 g/L) was added. Effluent was re-sampled after 48 h of activated carbon treatment to evaluate the potential for eluent to be regenerated.

### 2.3 Resin characterisation

#### 2.3.1 Particle size distribution

Particle size distribution of fresh, used, and regenerated resin was measured by laser diffraction using a Mastersizer 2000 Laser Diffraction Particle Analyzer (Malvern Panalytical, Spectris plc. UK) with a Hydro 2000MU sample dispersion unit in water. RI was set to 1.59 (polystyrene) with the weighted residual being 1–2% and obscuration ranging from 6 to 12%. Experiments were performed in triplicate.

#### 2.3.2 Scanning electron microscopy

Resin beads were oven dried at 50 °C overnight and sputter coated with a layer of platinum (∼10 nm). Images were taken on a SU8600 (Hitachi, Japan) at an accelerating voltage of 2 kV.

#### 2.3.3 BET surface area and pore size analysis

The specific surface area and pore characteristics of the samples were determined by nitrogen physisorption at 77 K using a Micromeritics TriStar II Plus analyzer (Version 3.04, Serial #3256). Prior to measurement, each sample was purified by washing three times with distilled water (25 mL each, 4 h with stirring at room temperature), followed by air drying for at least 24 h. The dried sample was then degassed overnight at 110 °C under nitrogen flow.

Nitrogen adsorption-desorption isotherms were recorded (in triplicate) for each sample. The Brunauer-Emmett-Teller (BET) surface area was calculated from the nitrogen adsorption data in the appropriate relative pressure range. Micropore surface area and volume were determined using the t-plot method. The average pore diameter was calculated using the 4 V/A by BET method.

### 2.4 Remediation of smoke tainted wine using a combined NF and solid phase adsorbent treatment

Three unoaked, smoke tainted wines, a Chardonnay, a rosé and a Cabernet Sauvignon (used in a previous study (Huo et al., 2025) were sourced from Cassegrain Wines (Port Macquarie, NSW, Australia). The wines (5 L per treatment, in duplicate) were fractionated using a Micro AA bench top membrane filtration system (VAF Memstar, Nuriootpa, SA, Australia; Fig. S1) equipped with a NF membrane (MWCO <1000 Da). The resulting retentate was passed through a cooling unit before being returned to the feed tank, while permeate was eluted through a fixed-bed column packed with adsorbent resin (BV = 200 mL) or an activated carbon cartridge (300 mL), each supplied by VAF Memstar. Treated permeate was then also returned to the feed tank. Wines were circulated through the combined NF and solid-phase adsorption process until a total of 5 L of permeate was generated and treated, after which, untreated and treated wines were sampled for compositional analysis and bottled (in 750 mL glass bottles, under screw cap) for sensory analysis (performed ∼6 weeks after bottling).

Five different remedial treatments were performed (in duplicate). The first two involved resin treatment of permeate derived from NF of the smoke tainted Chardonnay wine (5 L per replicate), using fresh and then regenerated resin (Table 2); with regeneration achieved via elution of 2% aqueous sodium hydroxide (5 BVs), 2% citric acid in 20% aqueous ethanol (5 BVs) and then water (3 BVs). For comparison, an activated carbon cartridge was then used to treat a third quantity of permeate derived from NF of the Chardonnay wine (5 L per replicate). Regenerated resin was then used to treat permeates derived from NF of the rosé and Cabernet Sauvignon wines (5 L per replicate). The NF operating conditions (i.e., adsorbent use, and permeate flow rates and volumes treated) are reported in Table 2.

**Table 2:** Experimental conditions for laboratory-scale NF and solid-phase adsorption treatment of smoke tainted Chardonnay, rosé and Cabernet Sauvignon wines.

Wine	Adsorbent	Permeate flow rate (BVs/h)	Volume of permeate treated (L)
Chardonnay 1	Fresh resin (BV = 200 mL)	7.5	5
Chardonnay 2	Regenerated resin (BV = 200 mL)	7.5	5
Chardonnay 3	Activated carbon (BV = 300 mL)	7.5	5
Rosé	Regenerated resin (BV = 200 mL)	7.5	5
Cabernet Sauvignon	Regenerated resin (BV = 200 mL)	4.2	5

BV = bed volume of adsorbent packed in the column.

### 2.5 Wine chemical analysis

#### 2.5.1 Basic wine chemistry

Wine pH and titratable acidity (TA, as g/L tartaric acid equivalents) were measured using a Mettler Toledo G20s compact titrator and automated sample changer (Port Melbourne, Vic., Australia). The alcohol content of wine (g/L) was measured using an Agilent 1100 high performance liquid chromatograph (HPLC) fitted with a reflective index detector (G1362A, Agilent Technologies, Forest Hill, Vic., Australia), following a previously published method (Li, Bindon, Bastian, Jiranek, & Wilkinson, 2017). The color density, hue and total phenolics of red and rosé wines were determined by the modified Somers color assay (Mercurio, Dambergs, Herderich, & Smith, 2007) using an Infinite® 200 PRO spectrophotometer (Tecan, Männedorf, Switzerland). White wine phenolics were estimated by measuring absorbance at 280 nm (Ortega, Lopez-Toledano, Mayen, Merida, & Medina, 2003), again with the Infinite® 200 PRO spectrophotometer but using Greiner UV star 96-well plates (Greiner Bio-One GmbH, Landsborough, Qld., Australia).

#### 2.5.2 Volatile phenols

Volatile phenols (guaiacol, 4-methylguaiacol, phenol, *o*-, *m*-, and *p*-cresol, syringol and 4-methylsyringol) were measured in wine and effluent samples using an Agilent 6890 gas chromatograph coupled to an Agilent 5973 mass spectrometer (Agilent Technologies, Forest Hill, Vic., Australia), following published stable isotope dilution assay (SIDA) methods (Hayasaka et al., 2013; Mirabelli-Montan et al., 2026). Briefly, samples (5 mL) were extracted with 2 mL of pentane/ethyl acetate (2:1) after addition of deuterium-labelled internal standards (i.e., *d*_4_-guaiacol, *d*_6_-phenol, *d*_5_-*o*-cresol and *d*_3_-syringol). High pH effluent samples were adjusted to wine pH (∼3.4) by addition of 10% aqueous citric acid, before addition of internal standards. ChemStation and MassHunter software (Agilent Technologies) were used for data acquisition and processing. Instrument operating conditions were as previously published (Mirabelli-Montan et al., 2026). Limits of detection were 1 μg/L (but lower values were recorded for data processing and visualization).

#### 2.5.3 Phenolic glycosides

Phenolic glycosides were measured (as syringol gentiobioside equivalents) in wine and effluent using an Agilent 1200 high-performance liquid chromatograph coupled to an AB SCIEX Triple Quad™ 4500 tandem mass spectrometer (Agilent Technologies) equipped with a Turbo V™ ion source (Framingham, MA, USA). Sample preparation for wine and low pH effluent followed a published SIDA method (Hayasaka et al., 2013; Mirabelli-Montan et al., 2026), with *d*_6_-syringol gentiobioside (i.e., the internal standard) spiked into samples prior to direct injection into the instrument. However, for effluent samples containing organic solvents, following the addition of internal standard, samples were concentrated to dryness using a Biotage TurboVap LV evaporator (Shimadzu, Rydalmere, NSW, Australia) before reconstitution in model wine, and then injection. For high pH effluent, samples were adjusted to wine pH (∼3.4, as above) before the addition of internal standard. Analyst software (AB SCIEX) was used for data acquisition and processing. Instrument operating conditions were as previously published (Mirabelli-Montan et al., 2026). Limits of detection were 1 μg/L (but lower values were again recorded for data processing and visualization).

### 2.6 Sensory analysis

The sensory profiles of untreated and treated wines were determined using the Rate-All-That-Apply (RATA) descriptive analysis method (Ares et al., 2014). Two sensory sessions were held (on different days), one for Chardonnay wines and one for rosé and Cabernet Sauvignon wines. Each RATA panel comprised 50 regular wine consumers, aged 23–83 years (with 20 males and 30 females for Chardonnay, and 18 males and 32 females for rosé and Cabernet Sauvignon). Sensory evaluation was conducted in a purpose-built sensory laboratory, under controlled environmental conditions, with wines (30 mL) served monadically in covered, 215 mL stemmed glasses, labelled with randomly generated 4-digit codes, and presented in a randomized order across participants. Before each sensory session, participants were introduced to the RATA procedure and a list of descriptors (Table S1) adapted from a previous study (Szeto et al., 2020). Participants rated the intensity of each attribute using 7-point scales (where 0 = not detected, 1 = “extremely low”, and 7 = “extremely high”). Codes, presentation order and data were generated with RedJade software (Redwood Shores, CA, USA). Panellists gave informed consent before participating in the study, which was approved by the Human Research Ethics Committee of Adelaide University (Ethics Approval No. H-2021-175).

### 2.7 Data analysis

Compositional data were analyzed by one-way analysis of variance (ANOVA) with mean comparisons conducted by Tukey's Honestly Significant Difference (HSD) post-hoc test at *p* ≤ 0.05 using XLSTAT (version 2022, Lumivero, Denver, CO, USA). Sensory data were analyzed by two-way ANOVA (using participants as a random factor and wines as a fixed factor) with mean comparisons carried out by Fischer's Least Significant Difference (LSD) test at *p* ≤ 0.05, using SenPAQ (v6, Qi Statistic Ltd., Ruscombe, Reading, UK). Principal Component Analysis (PCA) of statistically significant sensory data was also performed using XLSTAT.

## 3 Results and discussion

### 3.1 Desorption of free and glycosylated phenols during resin regeneration

**Fig. 1:**
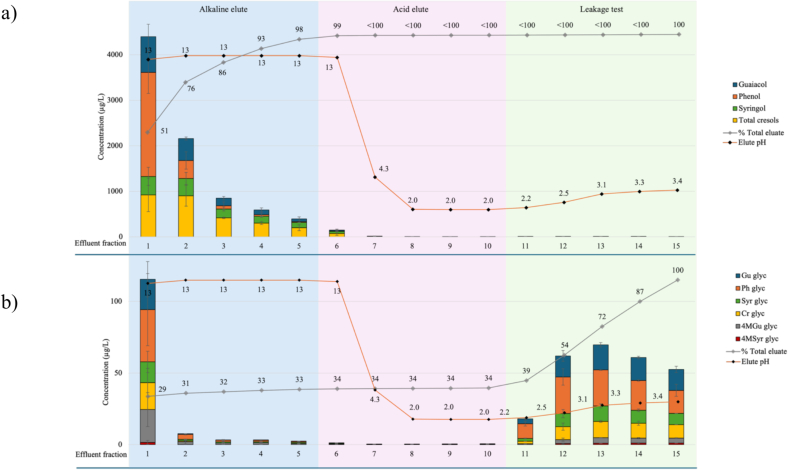
Concentration of (a) volatile phenols (μg/L) and (b) phenolic glycosides (μg/L) in column effluent (and cumulative percentage removal in total eluate,) during preliminary resin regeneration trial, involving elution with 2% aqueous sodium hydroxide (fractions 1 to 5, blue background), 2% aqueous citric acid (fractions 6 to 10, pink background), and model wine (fractions 11 to 15, green background). Data are means of two replicates (n = 2). Model wine = 12% aqueous ethanol saturated with KHT and pH adjusted to 3.4. The resin bed volume and the volume of each effluent fraction were 200 mL. Glyc = glycosides, Gu = guaiacol, 4MGu = 4-methylguaiacol, Cr = cresol, pH = phenol, Syr = syringol, 4MSyr = 4-methylsyringol. Phenolic glycosides were measured as syringol gentiobioside equivalents.

In a recent study, free and glycosylated phenols were successfully removed from a smoke tainted Chardonnay wine using a combined UF and solid phase adsorption treatment, thereby mitigating the perceived intensity of several smoke-related sensory attributes (Huo et al., 2025). However, despite regeneration of the resin, subsequent treatment of smoke tainted rosé and Cabernet Sauvignon wines resulted in carryover of phenolic glycosides; i.e., their concentrations increased significantly with remedial treatment (Huo et al., 2025). This was attributed to incomplete regeneration, such that residual phenolic glycosides remained on the resin. A preliminary regeneration trial was therefore conducted to establish the desorption of free and glycosylated phenols using an existing protocol involving elution with 2% aqueous sodium hydroxide, followed by 2% aqueous citric acid (to neutralize residual base), and then model wine to evaluate carryover of smoke taint compounds (Fig. 1).

The first effluent fraction collected during resin regeneration comprised 51% of the total pool of volatile phenols (∼4500 μg/L) eluted from the resin column (Figs. 1 and S2), with the following four effluent fractions accounting for a further 47% of volatile phenols. Approximately 150 μg/L (∼1%) of volatile phenols were eluted in fraction 6, which still comprised a significant proportion of sodium hydroxide based on its pH of 12.8. Low (< 7 μg/L) or non-detectable (< 1 μg/L) volatile phenol levels were observed in fractions 7 to 10 (i.e., when effluent pH was ≤4.3), suggesting desorption of volatile phenols was pH dependent. Small quantities of syringol and *p*-cresol (< 3 μg/L) were found in fractions 11 to 15 due to desorption by model wine (Figs. 1 and S2, Table S2). This indicated the potential for some carryover of volatile phenols, with any subsequent use of the regenerated resin.

Volatile phenols can deprotonate in alkaline solutions, i.e., when pH > pKa, giving rise to phenolates (phenoxides), which being ionized, are more soluble in aqueous solutions. This phenomenon has been exploited in previous studies, e.g., sodium hydroxide has been used to facilitate the extraction of phenols (Zhao & Lee, 2001) and thiols (Capone, Sefton, & Jeffery, 2011). When pH ≥ pKa + 2, an acid will be 99% deprotonated. Given phenol and the isomers of cresols have pKas between 9.95 and 10.28 (Aktaş, Şanlı, & Pekcan, 2006), alkaline solutions, including aqueous sodium carbonate (pH adjusted to 8, 10 or 12) were selected for further resin regeneration trials intended to evaluate the effect of pH on volatile phenol desorption.

Desorption of phenolic glycosides followed a very different trend to free volatile phenols (Figs. 1b and S3). Neither alkaline nor acidic eluents resulted in significant desorption of glycosylated phenols during resin regeneration; the observed elution of phenolic glycosides in fraction 1 (at 14–36 μg/L, being 29% of the total pool of phenolic glycosides eluted from the resin column) was attributed to residual wine in the column, rather than desorption by the alkaline eluent. Only when the resin was eluted with organic solvent (i.e., model wine), were phenolic glycosides desorbed (Figs. 1b and S3). Glycosylated phenols are not deprotonated in alkaline conditions, unlike free phenols. Their desorption during resin regeneration seemingly relies on the presence of a polar solvent rather than being pH dependent. Therefore, aqueous ethanol and isopropanol were selected for further resin regeneration trials, i.e., to evaluate the potential for alcoholic solutions to achieve desorption of phenolic glycosides.

Given the conversion of volatile phenols into phenolates in aqueous sodium hydroxide, the effect of pH on the quantification of volatile phenols in alkaline eluent was investigated. The recovery of deuterium-labelled internal standards (i.e., d_3_-syringol, d_4_-guaiacol, d_5_-*o*-cresol and d_6_-phenol) by direct extraction from 2% aqueous sodium hydroxide was significantly lower than recovery from 2% aqueous citric acid or model wine (Table S3). However, the expected quantity of deuterium-labelled internal standards was recovered when the sample was pH adjusted to ∼3.5 prior to extraction (Table S3). This can be explained by protonation of phenolates under acidic conditions, enabling extraction of volatile phenols. Importantly, there was no evidence of hydrolysis of internal standards during preparation of alkaline effluent samples (Table S3). The alkaline effluent samples collected during subsequent regeneration trials were therefore acidified before liquid-liquid extraction to ensure accurate quantitation of volatile phenols.

The HPLC-MS/MS method used to measure phenolic glycosides has only been validated for juice and wine (Hayasaka et al., 2013). Thus, to validate the method's application for analysis of effluent samples, the relative abundance of syringol gentiobioside (SyrGG, 50 μg/L injection) and its deuterium-labelled derivative (*d*_6_-SyrGG) were compared in model wine and in 2% aqueous sodium hydroxide (∼12 h after preparation, to evaluate the potential for hydrolysis to occur). Provided samples were adjusted to wine pH prior to instrumental analysis, relative abundance was found to be comparable between samples (Table S4); i.e., there was no evidence of hydrolysis. Acid hydrolysis can cleave glycosidic linkages, and phenolic glycosides have been shown to release their aglycones under strong acid hydrolysis conditions, i.e., at pH = 1.0, with heating to 100 °C for one hour (Kennison, Gibberd, Pollnitz, & Wilkinson, 2008), but hydrolysis does not occur under the mildly acid conditions employed in resin regeneration (i.e., 2% citric acid). As such, effluent samples from subsequent regeneration trials were pH adjusted to between 3.3 and 3.5 using aqueous sodium hydroxide or citric acid, as required.

### 3.2 Effect of eluent composition and pH on desorption of volatile phenols during resin regeneration

**Fig. 2:**
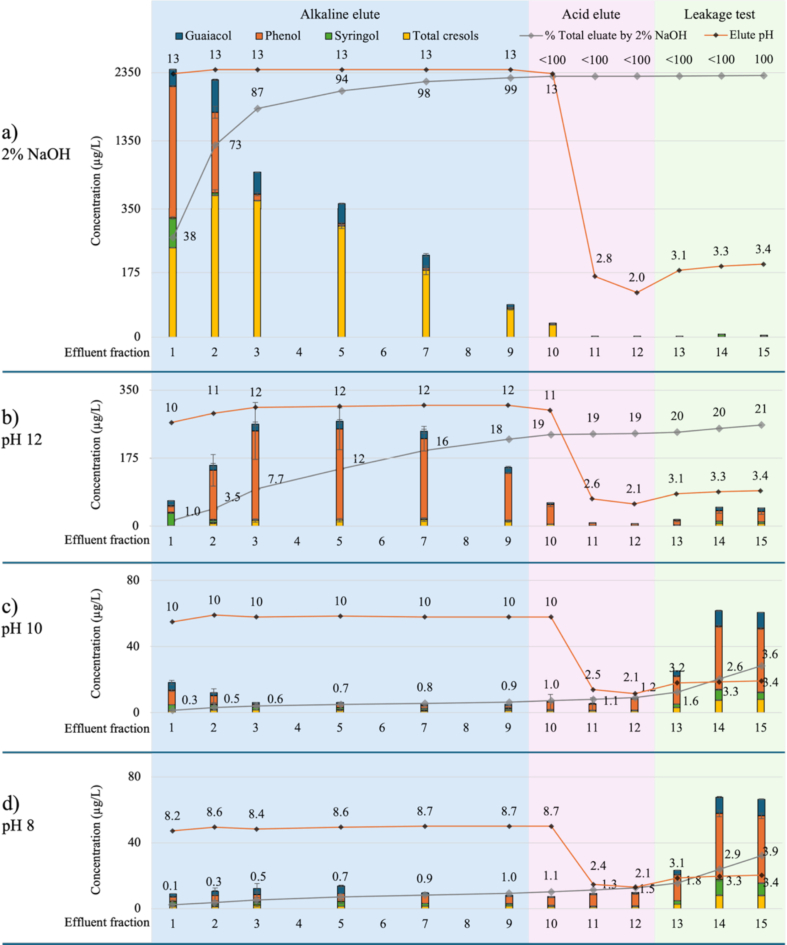
Concentration of volatile phenols (μg/L) in column effluent (and cumulative percentage removal relative to total elution achieved by 2% aqueous sodium hydroxide,) during resin regeneration trials, comparing elution with (a) 2% aqueous sodium hydroxide vs 5% aqueous sodium carbonate, pH adjusted to (b) 8, (c) 10 and (d) 12 (fractions 1 to 5, blue background), followed by 2% aqueous citric acid (fractions 10 to 12, pink background), and model wine (fractions 13 to 15, green background). Data are means of two replicates (n = 2). Model wine = 12% aqueous ethanol saturated with KHT and pH adjusted to 3.4. The resin bed volume and the volume of each effluent fraction were 50 mL.

Further regeneration trials were carried out to investigate the effect of pH on volatile phenol desorption. The desorption of volatile phenols by different alkaline solutions is shown in Figs. 2 and S4, and elution with 2% aqueous sodium hydroxide clearly resulted in greater desorption of volatile phenols than achieved in other treatments. Approximately 4600 μg/L of volatile phenols were eluted in fractions 1 and 2 (being ∼73% of the total pool of desorbed volatile phenols), with ∼94% of volatile phenols eluted in the first five effluent fractions, and a further ∼5% eluted by fraction 9 (Figs. 2a and S4a). Again, some volatile phenols were observed in fraction 10 (up to 33 μg/L, < 0.5%), likely due to the high aqueous sodium hydroxide content of effluent, with <1–2 μg/L observed once pH decreased <3.0. Only small quantities of syringol and cresol were observed in model wine (Fig. S4a), indicating negligible risk of any carryover.

Only 18% of the volatile phenols that were desorbed by 2% aqueous sodium hydroxide were eluted by 2% aqueous sodium carbonate at pH 12 (Fig. 2b), and when the pH of the sodium carbonate eluent was lowered (to 10 or 8), ≤ 1.0% volatile phenol desorption was achieved (Figs. 2c and 2d). This confirmed the importance of eluant pH being at least 2 units above the pKa of volatile phenols, to ensure their deprotonation and desorption, and therefore, the successful regeneration of the resin. Elution with 2% aqueous citric acid achieved little desorption of volatile phenols (Fig. 2), whereas model wine effluent (fractions 13 to 15) comprised volatile phenols at concentrations up to ∼40 μg/L (Fig. S4). The elution of phenolic glycosides was not considered as part of the pH trial, given they were only desorbed in meaningful quantities by aqueous alcoholic eluent.

### 3.3 Effect of eluent composition on desorption of phenolic glycosides during resin regeneration

The desorption of phenolic glycosides by effluent comprising aqueous ethanol or aqueous isopropanol (at 6% vs 12% vs 20%) was evaluated in a solvent-based resin regeneration trial (Table 1). Isopropanol was chosen as an alternative to ethanol because it is readily available in commercial quantities, and it is not subject to excise duty.

**Fig. 3:**
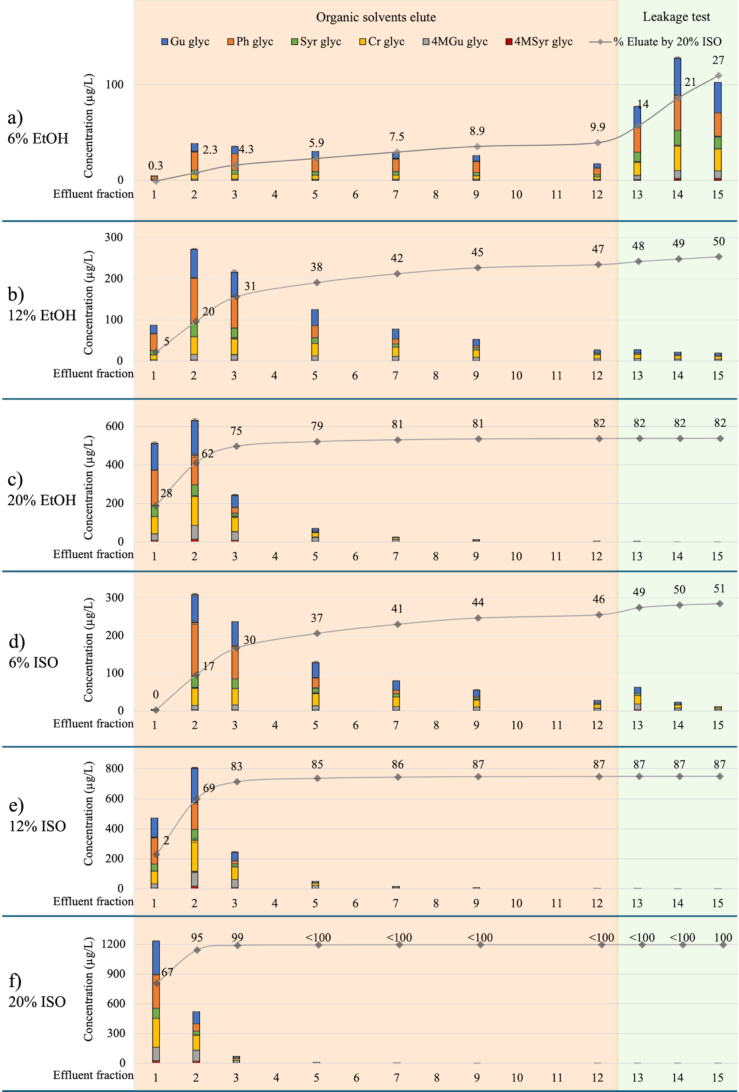
Concentration of phenolic glycosides (μg/L) in column effluent (and cumulative percentage removal relative to total elution achieved by 20% aqueous alcohol (ethanol (EtOH) or isopropanol (ISO),) during resin regeneration trials, comparing elution with (a) 6%, (b) 10% and (c) 20% aqueous ethanol or (d) 6%, (e) 10% and (f) 20% aqueous isopropanol (fractions 1 to 12, tan background), followed by model wine (fractions 13 to 15, green background). Data are means of two replicates (n = 2). Model wine = 12% aqueous ethanol saturated with KHT and pH adjusted to 3.4. The resin bed volume and the volume of each effluent fraction were 50 mL. Glyc = glycosides, Gu = guaiacol, 4MGu = 4-methylguaiacol, Cr = cresol, pH = phenol, Syr = syringol, 4MSyr = 4-methylsyringol. Phenolic glycosides were measured as syringol gentiobioside equivalents.

The concentration of glycosylated phenols in column effluent is shown in Figs. 3, S5 and S6. Elution with 20% aqueous isopropanol resulted in the greatest desorption of phenolic glycosides (Table S5), therefore other treatments were benchmarked against this eluent. A significant proportion (∼95%) of the total phenolic glycoside pool (∼1800 μg/L) eluted by 20% aqueous isopropanol was observed in the first two fractions of column effluent (Figs. 3 and S5c). After elution of 5 BVs of eluent, phenolic glycosides were only observed at ≤1 μg/L (accounting for <1% of the phenolic glycoside pool), including in model wine effluent (Figs. 3 and S5c), indicating successful regeneration, with no risk of carryover.

Similar results were achieved with 20% aqueous ethanol (Figs. 3c and S6c); 75% of the total phenolic glycoside pool desorbed by 20% aqueous isopropanol was desorbed in the first three effluent fractions. Phenolic glycosides were still being desorbed at detectable concentrations in fraction 12, but not in model wine fractions (Figs. 3c and S6c); 82% of the glycoconjugate pool was accounted for after elution of 15 fractions.

As the alcoholic strength of eluant decreased, so too did the desorption of phenolic glycoconjugates (Figs. 3, S5 and S6), regardless of the solvent used (ethanol or isopropanol). After elution of 12 fractions, 6%, 12% and 20% aqueous ethanol treatments accounted for 10%, 47% and 82% desorption of the phenolic glycoside pool eluted by 20% aqueous isopropanol, while 6% and 12% aqueous isopropanol achieved 46% and 87% desorption, respectively. At each alcoholic strength, isopropanol eluted a significantly higher proportion of the glycoconjugate pool than ethanol (Table S5). Importantly, detectable levels of phenolic glycosides were observed in model wine effluent following resin regeneration with 6% aqueous isopropanol (Fig. S5a), and 6% or 12% aqueous ethanol (Figs. S6a and S6b), indicating some risk of carryover, especially where <12 BVs of eluent is used.

It is well known that the physical properties of polymeric materials can vary (due to shrinkage or swelling) under different conditions, e.g., in the presence of different solvents (Kappert et al., 2019; Sienkiewicz, Krasucka, Charmas, Stefaniak, & Goworek, 2017). In the current study, a decrease in volume was observed when resin was soaked in 2% aqueous sodium hydroxide and an increase observed in aqueous isopropanol (Fig. S7). The expansion of the resin may expose more binding sites to the effluent, enhancing desorption of adsorbates. When the polarity indices of water, ethanol and isopropanol (being 100, 5.2 and 3.9, respectively) are considered, it can be seen that elution of phenolic glycosides may depend on solvent polarity; i.e., desorption seemingly occurred more readily when solvent polarity was lower.

From a commercial perspective, the cost, environmental impact and flammability of solvent systems used for resin regeneration need to be taken into consideration. Ethanol is a byproduct of some winemaking processes, e.g., distillation employed in the production of no and low alcohol wines (Schmidtke, Blackman, & Agboola, 2012) and at ≤20% ethanol there is no flammability concern. As such, 20% aqueous ethanol was used for subsequent resin regeneration.

Similar trends were observed for the elution of volatile phenols by aqueous alcoholic solutions; the higher the alcoholic strength of eluent (ethanol or isopropanol), the higher the effluent volatile phenol concentration (Tables S6 and S7). However, none of the aqueous alcoholic eluents achieved volatile phenol desorption rates comparable to that achieved by 2% aqueous sodium hydroxide (Table S8). For example, eluting 12 BVs of 6% aqueous ethanol only achieved desorption of 4% of the volatile phenols eluted by 2% aqueous sodium hydroxide, with 12% and 20% aqueous ethanol eluting ∼9% and 19% of the volatile phenol pool eluted with 2% aqueous sodium hydroxide (Table S7). Isopropanol again performed better than ethanol (Tables S6, S7 and S8). Importantly, none of the aqueous alcoholic eluents prevented carryover of volatile phenols; in all cases, model wine effluent was contaminated with residual volatile phenols due to carryover (Tables S6 and S7).

### 3.4 Optimization of eluent composition for resin regeneration

Regeneration trials established the most effective eluents for desorption of free and glycosylated phenols from the adsorbent resin were 2% aqueous sodium hydroxide, followed by 2% aqueous citric acid and 20% aqueous alcoholic solvent (either ethanol or isopropanol). A final trial aimed at improving the efficiency of the regeneration protocol was conducted: (i) comparing volatile phenol desorption using 2% vs 5% aqueous sodium hydroxide (9 BVs); and (ii) evaluating phenolic glycoside desorption using 2% citric acid in 20% aqueous ethanol (6 BVs; Table 1). The potential for activated carbon to remove smoke taint compounds from low pH aqueous ethanol effluent, enabling its regeneration and reuse was also investigated.

**Fig. 4:**
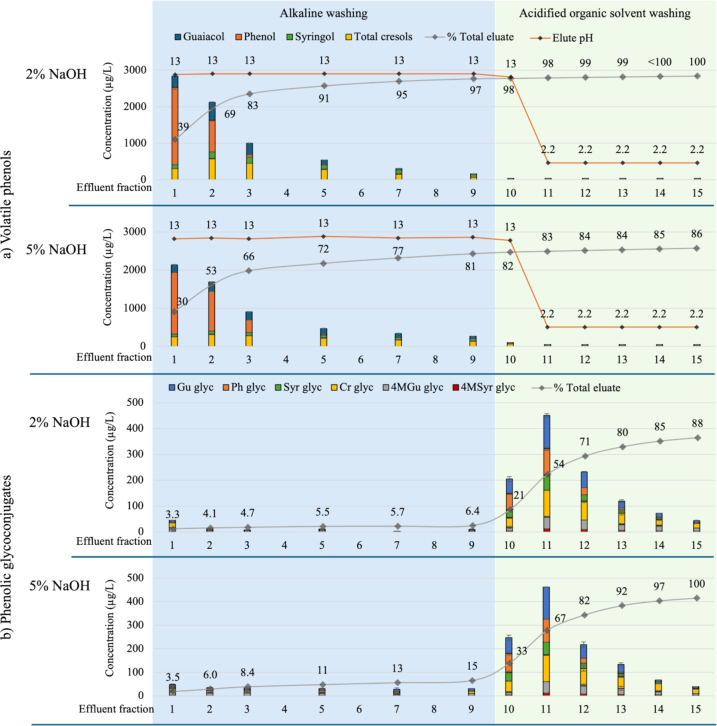
Concentration of (a) volatile phenols (μg/L) or (b) phenolic glycosides (μg/L) in column effluent (and cumulative percentage removal in total eluate,) during resin regeneration trials, comparing elution with 2% vs 5% aqueous sodium hydroxide (fractions 1 to 9, blue background), followed by 2% citric acid in 20% aqueous ethanol (fractions 10 to 15, green background). Data are means of two replicates (n = 2). The resin bed volume and the volume of each effluent fraction were 50 mL. Glyc = glycosides, Gu = guaiacol, 4MGu = 4-methylguaiacol, Cr = cresol, pH = phenol, Syr = syringol, 4MSyr = 4-methylsyringol. Phenolic glycosides were measured as syringol gentiobioside equivalents.

Desorption of free and glycosylated phenols is shown in Figs. 4, S8 and S9, and Tables S9 and S10. Surprisingly, with the exception of phenol, elution with 2% aqueous sodium hydroxide resulted in significantly higher volatile phenol desorption than with 5% aqueous sodium hydroxide (Table S9). Thus, 2% aqueous sodium hydroxide was retained as the optimal alkaline eluent for desorption of volatile phenols. The first column fraction eluted almost 3000 μg/L of volatile phenols, accounting for 39% of the total volatile phenol pool eluted, with 97% volatile phenol desorption achieved after elution of 9 BVs (Figs. 4a and S8). In contrast, only 30% and 81% of volatile phenols were desorbed after elution of 1 and 9 BVs of 5% aqueous sodium hydroxide (Figs. 4a and S8). During elution with acidified 20% aqueous ethanol (i.e., fractions 10 to 15), lower volatile phenol levels were observed in effluent from 2% aqueous sodium hydroxide treated resin than from 5% aqueous sodium hydroxide treated resin (Figs. 4a and S8). These results further confirmed the decreased efficacy of 5% vs 2% aqueous sodium hydroxide eluent for volatile phenol desorption.

The stronger alkaline eluent was expected to increase the solubility, and therefore desorption, of volatile phenols through deprotonation. However, desorption of volatile phenols from resin decreased under more alkaline conditions. This was nevertheless, in agreement with previous studies. When aqueous sodium hydroxide was used to facilitate the extraction of 3-mercaptohexan−1-ol from wine, a similar decrease in extraction efficacy was observed when the base concentration was above 2 M or 8% (Capone et al., 2011). Decreased enrichment of 2,4-dimethylphenol, 2,3,5-trimethylphenol and 2,4-dichlorophenol in water was also reported when the concentration of the acceptor phase (sodium hydroxide) exceeded 0.5 M (2%), during analysis of phenols by HPLC (Zhao et al., 2001). This was attributed to a salting-out effect (Yabroff, 1940) and was consistent with results from a solubility study (Noubigh, Abderrabba, & Provost, 2007), whereby ferulic and syringic acid solubility in water decreased with the addition of chloride salts (*T* = 298.15 K). Hence, 2% aqueous sodium hydroxide was retained as the optimal alkaline eluent.

The addition of citric acid to the 20% aqueous ethanol eluent achieved neutralization of residual base (Fig. 4a), alongside desorption of phenolic glycosides from the adsorbent resin (Fig. 4b). Some glycoconjugates were desorbed by the alkaline eluent, but fraction 11 had the highest phenolic glycoside concentrations, after which effluent glycoconjugate concentrations progressively declined to <20 μg/L by the end of regeneration (i.e., in fraction 15; Figs. 4b and S9). Although the stronger 5% alkaline eluent desorbed a higher proportion of phenolic glycosides (than the 2% alkaline eluent), the effluent compositions of fractions 10 to 15 were not statistically significant (Table S10). In a previous study, a double extraction of *m*-cresol from an organic layer was achieved by eluting with 0.1 M hydrochloric acid, with the addition of salts to the donor phase (Zhao et al., 2001), again, presumably attributable to a salting out effect. However, studies into the effect of salts on the solubility of glycosides in organic solvent systems are limited. Further research is therefore warranted to investigate any salting out effect on the desorption of glycoconjugates from resin.

Research findings demonstrated that to successfully regenerate the adsorbent resin, the use of a highly alkaline solution (i.e., at least 5 BVs of 2% aqueous sodium hydroxide) and a polar aqueous alcoholic solution (i.e., at least 5 BVs of 20% aqueous ethanol) should be included in the regeneration protocol. Three BVs of 2% citric acid (in water or 20% aqueous ethanol) neutralized the column, ensuring no carryover of base into wine following resin regeneration.

Improper disposal of organic solvent can be harmful to the environment, thus regeneration of low pH aqueous ethanol effluent (i.e., fractions 11 to 15) was attempted via treatment with activated carbon. The initial concentrations of free and glycosylated phenols in the combined ethanolic effluent were 32–49 and 171–186 μg/L, respectively, and concentrations decreased substantially, to <5 and 50 μg/L, respectively, following activated carbon treatment (Table S11). This demonstrated the feasibility of regenerating and reusing aqueous alcoholic solutions, albeit, in a commercial setting, passing eluent through an activated carbon column would likely be a more efficient approach to regeneration.

### 3.5 Morphological and pore analysis of resin

**Fig. 5:**
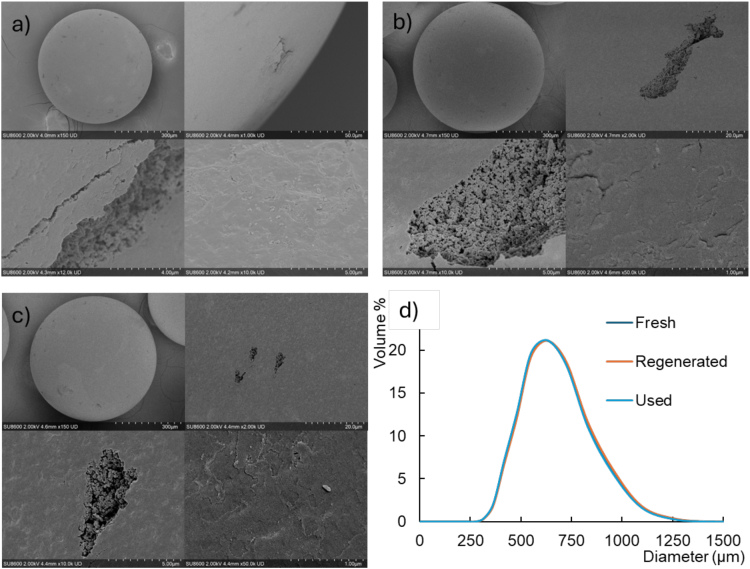
SEM analysis of (a) fresh, (b) used, and (c) regenerated adsorbent resin, and corresponding (d) particle size distribution measured by laser diffraction.

Scanning electron microscopy (SEM) images of the polystyrene-based adsorber beads (Fig. 5) revealed no substantial morphological differences among the fresh (Fig. 5a), used (Fig. 5b), and regenerated (Fig. 5c) samples. All beads exhibited a predominantly smooth outer surface interspersed with minor cracks, which exposed an internal pore structure resembling a popcorn-like morphology, which is typical for cross-linked polystyrene-divinylbenzene resins. The fresh resin appeared marginally smoother at higher magnifications, potentially due to the absence of residual adsorbates or minor surface fouling. These observations suggest that the adsorption process for extracting smoke-derived phenolic compounds from wine and subsequent regeneration do not induce significant physical damage or alteration to the bead structure.

The particle size distribution was nearly identical across all samples, with median particle diameters of 579.3 ± 4.4, 577.9 ± 3.8, and 585.0 ± 3.4 μm obtained for fresh, used and regenerated resin beads, respectively (Fig. 5d). These values fall within the measurement uncertainty, indicating no appreciable fragmentation, swelling, or attrition during use or regeneration. This stability is crucial for maintaining consistent flow dynamics and adsorption efficiency in packed-bed systems, supporting the resin's suitability for repeated cycles in wine treatment applications.

Nitrogen physisorption analysis was conducted to evaluate the surface area and pore characteristics of the resins, as these parameters directly influence adsorption capacity for phenolic compounds. The results, summarized in Table 3, show a decrease in BET surface area from 1093 ± 50 m^2^/g in the fresh resin to 902 ± 96 m^2^/g in used resin, representing a ∼ 17% reduction. Similarly, the t-plot micropore area and volume both declined by ∼18% with resin use. These reductions are attributable to pore blockage by adsorption of volatile phenols and phenolic glycosides, and other wine constituents, which occupy active sites and thus, restrict nitrogen access during measurement. Upon regeneration, the BET surface area recovered to 927 ± 29 m^2^/g, a partial restoration that suggests effective but incomplete removal of foulants, possibly due to strong binding of some wine compounds. Although the regeneration protocol effectively prevents carry-over of smoke taint compounds, it does not fully restore the adsorption capacity; consequently, a gradual decrease in the resin's ability to remove smoke taint compounds from wine may occur with prolonged reuse.

**Table 3:** BET surface area, micropore area, micropore volume, and average pore diameter of fresh, used, and regenerated adsorbent resin samples (mean ± standard deviation, *n* = 3).

	BET surface area (m^2^/g)	t-plot micropore area (m^2^/g)	t-plot micropore volume (cm^3^/g)	Average pore diameter (Å)
Fresh resin	1093 ± 50	809 ± 37	0.319 ± 0.014	19.3 ± 0.03
Used resin	902 ± 96	663 ± 69	0.262 ± 0.027	19.6 ± 0.06
Regenerated resin	927 ± 29	689 ± 22	0.271 ± 0.008	19.3 ± 0.029

Data are means of three replicates (*n* = 3) ± standard deviation.

The average pore diameter remained consistent across all samples (at ∼19.3–19.6 Å), with variations well within experimental error. This invariance corroborates the SEM findings, indicating that the pore network undergoes no irreversible structural changes, such as collapse or widening, during the adsorption-regeneration cycle.

### 3.6 Remediation of smoke tainted wine using a combined NF and solid phase adsorbent treatment

To demonstrate the efficacy of the optimized resin regeneration protocol, smoke tainted wines were remediated using a combined NF and solid phase adsorption treatment, with regeneration of the adsorbent resin between treatments. An activated carbon cartridge was included for comparison (i.e., as the benchmark for amelioration of smoke taint).

Changes in free and glycosylated phenols during treatment are reported in Table 4. Wine volatile phenols progressively decreased with treatment (by as much as 42–59%, and ∼ 50% overall), indicating the resin did not reach saturation. This was also supported by the low concentration of volatile phenols observed in effluent sampled during remediation experiments (Table S12). Furthermore, there were no significant differences in the volatile phenol profiles of the three treated Chardonnay wines, demonstrating resin performance (both fresh and regenerated) was comparable to that of the activated carbon cartridge. It might nevertheless be worth comparing resin performance against other adsorbents, e.g., molecularly imprinted polymers (Huo et al., 2024) in future studies.

**Table 4:** Changes in concentration of free and glycosylated phenols (μg/L) in smoke tainted Chardonnay (Ch), rosé and Cabernet Sauvignon (CS) wines during remediation using a combined NF and adsorbent resin treatment, with their percentage removal determined at intervals compared to untreated wines shown in italics.

Wine	Volume of permeate treated (mL)	Gu		4MGu		*o*-Cr		*m*-Cr		*p*-Cr		Ph		Syr		4MSyr		Gu glyc		4MGu glyc		Ph glyc		Cr glyc		Syr glyc		4MSyr glyc		Total glyc	
Untreated Ch	0	43 a		11 a		6.9 a		18 a		15 a		61 a		62 a		18 a		703		268		809 abc		610		1067		165		3012	
	1000	37 bc	*15%*	9.2 b	*16%*	5.8 bc	*16%*	15 bc	*17%*	13 bc	*15%*	53 ab	*14%*	54 bc	*14%*	16 abc	*12%*	693	*1%*	268	*0%*	821 abc	*−1%*	602	*1%*	1083	*−1%*	169	*−2%*	3034	*−1%*
	2000	31 bcde	*27%*	7.9 d	*28%*	5.0 cd	*28%*	14 bcd	*26%*	11 bcd	*24%*	46 bc	*25%*	47 bcd	*24%*	14 bcd	*23%*	691	*2%*	265	*1%*	813 abc	*0%*	594	*3%*	1064	*0%*	167	*−1%*	3001	*0%*
Treated Ch (fresh resin)	3000	28 def	*34%*	7.1 e	*35%*	4.5 def	*36%*	12 def	*35%*	9.9 def	*33%*	41 cd	*33%*	41 de	*34%*	12 de	*32%*	660	*6%*	259	*3%*	797 abc	*1%*	576	*6%*	1094	*−3%*	169	*−2%*	2979	*1%*
	4000	25 efgh	*42%*	6.1 e	*44%*	3.9 fgh	*43%*	10 efgh	*43%*	8.9 efg	*40%*	36 cde	*42%*	38 defg	*40%*	11 defg	*38%*	718	*−2%*	271	*−1%*	833 abc	*−3%*	587	*4%*	1115	*−4%*	172	*−4%*	3109	*−3%*
	5000	22 fgh	*49%*	5.4 e	*50%*	3.6 fghi	*49%*	9.4 fgh	*49%*	7.9 fgh	*47%*	32 de	*48%*	33 efg	*47%*	10 efg	*46%*	684	*3%*	271	*−1%*	817 abc	*−1%*	598	*2%*	1094	*−3%*	170	*−3%*	3036	*−1%*
	1000	38 ab	*12%*	9.5 c	*13%*	6.1 b	*12%*	16 b	*13%*	13 ab	*12%*	53 ab	*14%*	55 abc	*11%*	17 abc	*10%*	713	*−1%*	280	*−5%*	854 abc	*−6%*	636	*−4%*	1109	*−4%*	174	*−5%*	3129	*−4%*
	2000	33 bcd	*24%*	8.3 c	*24%*	5.1 cd	*26%*	14 bcd	*25%*	11 bcd	*23%*	46 bc	*25%*	48 bcd	*24%*	14 bcd	*22%*	672	*4%*	267	*0%*	822 abc	*−2%*	591	*3%*	1062	*0%*	166	*−1%*	2989	*1%*
Treated Ch (regenerated resin)	3000	27 def	*37%*	6.7 c	*39%*	4.4 def	*37%*	12 def	*37%*	9.6 defg	*35%*	38 cde	*39%*	39 def	*38%*	12 def	*36%*	686	*2%*	265	*1%*	807 abc	*0%*	594	*3%*	1092	*−2%*	173	*−5%*	3022	*0%*
	4000	24 fgh	*45%*	5.7 c	*48%*	3.7 fghi	*47%*	9.9 fgh	*46%*	8.4 efgh	*44%*	32 de	*48%*	34 efg	*45%*	10 efg	*45%*	727	*−3%*	276	*−3%*	897 a	*−11%*	645	*−6%*	1120	*−5%*	178	*−8%*	3198	*−6%*
	5000	20 gh	*54%*	4.9 cd	*56%*	3.1 hi	*55%*	8.4 gh	*54%*	7.3 gh	*51%*	27 e	*55%*	29 fg	*54%*	8.8 fg	*52%*	708	*−1%*	274	*−2%*	879 ab	*−9%*	630	*−3%*	1136	*−7%*	179	*−8%*	3176	*−5%*
	1000	37 bc	*14%*	9.4 cd	*14%*	5.8 bc	*17%*	15 bc	*17%*	13 bc	*15%*	54 ab	*12%*	57 ab	*9%*	17 ab	*7%*	746	*−6%*	287	*−7%*	894 a	*−10%*	645	*−6%*	1160	*−9%*	178	*−8%*	3265	*−8%*
	2000	31 cde	*28%*	7.7 cd	*29%*	4.9 cde	*29%*	13 cde	*30%*	10 cde	*30%*	44 bc	*28%*	47 cd	*25%*	14 cd	*24%*	695	*1%*	267	*0%*	786 bc	*3%*	607	*1%*	1108	*−4%*	173	*−5%*	3029	*−1%*
Treated Ch (activated carbon)	3000	26 defg	*39%*	6.6 cd	*39%*	4.1 efg	*41%*	11 defg	*40%*	8.6 efg	*42%*	37 cde	*40%*	39 de	*37%*	12 def	*35%*	673	*4%*	261	*2%*	753 c	*7%*	580	*5%*	1088	*−2%*	166	*0%*	2941	*2%*
	4000	22 fgh	*49%*	5.6 cd	*49%*	3.4 ghi	*50%*	9.2 fgh	*50%*	7.5 gh	*50%*	32 de	*48%*	34 efg	*46%*	10 efg	*44%*	644	*8%*	256	*4%*	783 bc	*3%*	557	*9%*	1074	*−1%*	168	*−1%*	2924	*3%*
	5000	18 h	*58%*	4.5 cd	*59%*	2.8 i	*59%*	7.8 h	*58%*	6.2 h	*58%*	27 e	*56%*	28 g	*55%*	8.5 g	*54%*	701	*0%*	265	*1%*	792 bc	*2%*	574	*6%*	1130	*−6%*	175	*−6%*	3064	*−2%*
*P*		*<0.0001*		*<0.0001*		*<0.0001*		*<0.0001*		*<0.0001*		*<0.0001*		*<0.0001*		*<0.0001*		*0.142*		*0.897*		*0.001*		*0.082*		*0.369*		*0.570*		*0.095*	
Untreated Rosé	0	26 a		2.9 a		3.4 a		9.0 a		9.0 a		39 a		17 a		3.2 a		395		79		365		237		118		13 b		971	
	1000	23 b	*13%*	2.6 b	*12%*	3.1 a	*11%*	7.9 b	*12%*	8.0 b	*11%*	35 b	*12%*	15 b	*11%*	2.8 ab	*12%*	428	*−8%*	84	*−5%*	375	*−3%*	242	*−2%*	125	*−5%*	14 ab	*−6%*	1025	*−6%*
	2000	20 c	*24%*	2.3 c	*22%*	2.6 b	*25%*	6.8 c	*24%*	6.8 c	*24%*	29 c	*26%*	13 c	*21%*	2.4 bc	*27%*	435	*−10%*	86	*−8%*	380	*−4%*	253	*−6%*	128	*−8%*	14 ab	*−7%*	1043	*−7%*
Treated rosé (regenerated resin)	3000	17 d	*35%*	2.0 d	*33%*	2.3 bc	*33%*	6.0 d	*33%*	6.2 c	*32%*	25 d	*36%*	12 d	*28%*	2.1 cd	*36%*	432	*−9%*	86	*−8%*	387	*−6%*	248	*−4%*	128	*−8%*	15 ab	*−10%*	1047	*−8%*
	4000	15 e	*44%*	1.6 e	*44%*	2.0 cd	*40%*	5.1 e	*43%*	5.4 d	*41%*	21 e	*46%*	11 e	*37%*	1.8 d	*46%*	419	*−6%*	83	*−5%*	362	*1%*	238	*0%*	131	*−11%*	15 ab	*−12%*	1011	*−4%*
	5000	13 f	*51%*	1.4 f	*51%*	1.8 d	*49%*	4.4 f	*51%*	4.5 e	*50%*	18 f	*54%*	9.6 f	*43%*	1.5 d	*53%*	408	*−3%*	83	*−5%*	376	*−3%*	249	*−5%*	133	*−12%*	16 a	*−18%*	1016	*−5%*
*P*		*<0.0001*		*<0.0001*		*<0.0001*		*<0.0001*		*<0.0001*		*<0.0001*		*<0.0001*		*0.000*		*0.331*		*0.231*		*0.462*		*0.609*		*0.094*		*0.061*		*0.387*	
Untreated CS	0	43 a		5.7 a		13 a		17 a		18 a		88 a		52 a		4.1 a		316		86		417		191		202		26		1046	
	1000	37 b	*13%*	5.0 b	*13%*	11.2 b	*12%*	14.9 b	*13%*	16 b	*10%*	76 b	*13%*	47 b	*10%*	3.9 b	*5%*	321	*−2%*	88	*−3%*	431	*−4%*	206	*−8%*	204	*−1%*	28	*−6%*	1072	*−2%*
	2000	32 b	*25%*	4.3 b	*24%*	9.8 b	*23%*	13.5 b	*22%*	14 b	*22%*	66 b	*25%*	41 b	*21%*	3.4 b	*17%*	317	*0%*	87	*−2%*	406	*3%*	200	*−5%*	200	*1%*	28	*−7%*	1039	*1%*
Treated CS (regenerated resin)	3000	28 b	*33%*	3.9 b	*33%*	8.8 b	*31%*	12.3 b	*29%*	12 b	*32%*	58 b	*34%*	37 b	*29%*	3.1 b	*25%*	325	*−3%*	89	*−4%*	434	*−4%*	204	*−7%*	211	*−4%*	28	*−8%*	1088	*−4%*
	4000	25 c	*42%*	3.5 c	*39%*	8.0 c	*37%*	10.8 c	*37%*	10.9 c	*40%*	50 c	*42%*	33 c	*37%*	2.7 c	*33%*	327	*−3%*	92	*−8%*	425	*−2%*	210	*−10%*	210	*−4%*	30	*−13%*	1083	*−4%*
	5000	21 c	*50%*	3.0 bc	*48%*	6.8 c	*46%*	9.2 c	*46%*	9.4 c	*48%*	43 c	*61%*	29 c	*44%*	2.4 bc	*42%*	320	*−1%*	90	*−5%*	429	*−3%*	206	*−7%*	209	*−3%*	29	*−9%*	1075	*−3%*
*P*		*<0.0001*		*<0.0001*		*<0.0001*		*<0.0001*		*0.000*		*<0.0001*		*<0.0001*		*0.000*		*0.974*		*0.840*		*0.827*		*0.688*		*0.817*		*0.562*		*0.907*	

Data are means of two replicates (n = 2).

Different letters (within columns, for each wine) indicate statistical significance (p ≤ 0.05, one-way ANOVA).

Phenolic glycosides were measured as syringol gentiobioside equivalents.

Gu = guaiacol; 4MGu = 4-methylguaiacol; Cr = cresol; pH = phenol; Syr = syringol; 4MSyr = 4-methylsyringol; glyc = glycosides.

Given the use of NF for wine fractionation, there was little permeation of phenolic glycosides (< 15% of the glycoconjugate pool present in untreated wines was observed in permeate, Table S12), in agreement with previous findings (Huo et al., 2025). This accounts for the similar phenolic glycoside concentrations being observed in treated wines, rather than resin saturation. The column effluent consistently comprised lower free and glycosylated phenol concentrations than untreated wine and NF permeate (Table S12), demonstrating there was no carryover of smoke taint compounds. This finding confirmed the efficacy of the NF membrane and the regeneration protocol (i.e., the use of acidified aqueous ethanol) in rejecting and desorbing glycoconjugates, respectively.

**Fig. 6:**
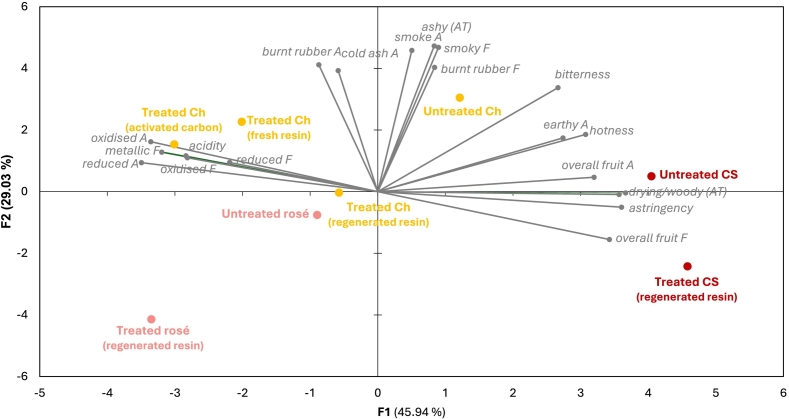
PCA biplot of mean sensory attribute ratings for smoke tainted Chardonnay (Ch), rosé and Cabernet Sauvignon (CS) wines before and after remediation using a combined NF and adsorbent resin treatment. A = aroma; F = flavor; AT = aftertaste.

Sensory analysis of treated wines confirmed the intensity of several smoke-related attributes decreased, in particular ratings for smoke aroma and smoky flavor (Fig. 6, Table S13). Resin treated Chardonnay wines also exhibited a less intense ashy aftertaste, and decreased bitterness, astringency and hotness, while activated carbon treatment impacted fruit expression and introduced some oxidized notes. Some loss of fruit expression was also perceived in treated rosé wine, but not in treated Cabernet Sauvignon wine.

The biplot generated from PCA of sensory data (Fig. 6) demonstrated separation of Chardonnay wines along the *x-axis* due to decreased fruit and smoke aromas and flavors, and increased oxidized and/or reduced characters resulting from membrane filtration and solid-phase adsorption treatments. The Cabernet Sauvignon wines were positioned furthest to the right reflecting their higher fruit expression compared to rosé and Chardonnay wines. Treated and untreated Cabernet Sauvignon were separated along the *y-axis* due to the decreases in cold ash aroma and smoky flavor achieved with remediation. Treated rosé was similarly positioned away from untreated rosé, due to decreased fruit and earthy aromas, smoke aroma and flavor, and ashy aftertaste.

The only significant differences in basic wine chemistry measures were TA, ethanol and total phenolics (absorbance at A280) among Chardonnay wines (Table S14); the basic composition of rosé and Cabernet Sauvignon wines was not affected by remedial treatments. The activated carbon cartridge resulted in removal of a significantly higher proportion of phenolic compounds from the Chardonnay wine than the resin (Table S14). No loss of phenolic compounds or anthocyanins was observed following treatment of rosé and Cabernet Sauvignon wines (Table S14), demonstrating their retention as a consequence of NF prior to resin treatment.

The effects of NF and solid-phase adsorption on other desirable wine constituents (e.g., aroma volatiles) were not investigated but may have explained the loss of fruit expression in some treated wines, similar to that observed in other studies (Huo et al., 2024). The scope of the current study did not include evaluation of membrane fouling (Du, Shi, Jegatheesan, & Haq, 2020; Sui et al., 2022) or resin fouling (Abrams, 1982; H. Li, Li, Shuang, Zhou, & Li, 2014), but these phenomena may warrant consideration when remedial treatments are applied at commercial scale.

## 4 Conclusion

The present study investigated the regeneration of an adsorbent resin used in the amelioration of smoke tainted wine. A preliminary regeneration trial confirmed the carryover of phenolic glycosides due to incomplete regeneration of resin by aqueous sodium hydroxide. Subsequent trials demonstrated the influence of pH and alcoholic strength on the desorption of free and glycosylated volatile phenols from resin, enabling the resin regeneration protocol to be optimized. Eluting resin with at least 5 BVs of 2% aqueous sodium hydroxide, 3 BVs of 2% aqueous citric acid and 5 BVs of 20% aqueous ethanol or aqueous isopropanol achieved adequate regeneration, such that there was no risk of smoke taint compounds being carried over between remedial treatments. The protocol was further modified to combine neutralization and desorption of volatile phenol glycoconjugates, to improve process efficiency. The optimized protocol was then demonstrated during the amelioration of three smoke tainted wines, using a combined NF and solid phase adsorption treatment. Chemical and sensory analysis of treated wines confirmed mitigation of smoke taint with no carryover of smoke taint compounds between wines. The combined use of NF and adsorbent resin does not resolve the presence of phenolic glycosides. As such, future research could investigate the potential for UF and NF membranes to be used sequentially to isolate a fraction enriched in phenolic glycosides for subsequent treatment (e.g., addition of hydrolytic enzymes and then adsorbent treatment). Analysis of the adsorbent resin morphology and pore structure indicated that the resin beads are structurally stable, exhibiting no major physical changes after use or regeneration. However, the regeneration protocol is unable to fully restore the accessible surface area, which could lead to progressive fouling in the long-term. The potential for diminished sorptive efficacy due to resin fouling may also warrant further research. Molecular-level analyses could also be employed to elucidate both the mechanisms underpinning analyte adsorption-desorption, and any effects of repeated regeneration on adsorbent binding capacity. Finally, whilst the cost of the adsorbent resin is comparable to that of the activated carbons typically used for remediation of smoke taint, there are obviously additional costs associated with the use of resin in combination with membrane filtration. This approach nevertheless offers other important benefits, being decreased wine volume and quality losses and need for costly waste disposal, that would otherwise be incurred with activated carbon treatment.

## CRediT authorship contribution statement

**Yiming Huo:** Writing – original draft, Visualization, Validation, Investigation, Formal analysis, Data curation, Conceptualization. **Renata Ristic:** Writing – review & editing, Visualization, Supervision, Resources, Formal analysis, Conceptualization. **David Wollan:** Writing – review & editing, Resources, Project administration, Methodology, Funding acquisition, Conceptualization. **Manuella Cazelato Pires:** Writing – review & editing, Resources, Investigation, Formal analysis, Data curation. **Lukas Gerstweiler:** Writing – review & editing, Visualization, Supervision, Resources, Methodology, Data curation, Conceptualization. **Richard Muhlack:** Writing – review & editing, Visualization, Supervision, Resources, Conceptualization. **Markus Herderich:** Writing – review & editing, Supervision, Resources, Funding acquisition, Conceptualization. **Kerry Wilkinson:** Writing – review & editing, Writing – original draft, Visualization, Supervision, Resources, Project administration, Methodology, Funding acquisition, Formal analysis, Data curation, Conceptualization.

## Declaration of competing interest

The authors declare that they have no known competing financial interests or personal relationships that could have appeared to influence the work reported in this paper.

## Data Availability

Data will be made available on request.
