# Supplementary material for: Optimizing the use of adsorbent resin for the amelioration of smoke tainted wine
Source: Food Chem X. 2026 Mar 19;35:103772. doi: 10.1016/j.fochx.2026.103772 (PMC13081679; doi:10.1016/j.fochx.2026.103772)
Supplement: Supplementary material [file mmc1.docx]

**Supplementary Material for**

**Optimizing the use of adsorbent resin for the amelioration of smoke tainted wine**

Yiming Huo ^a,b^, Renata Ristic ^a,b^, David Wollan ^c^, Manuella Cazelato Pires ^d^, Lukas Gerstweiler ^d^, Richard Muhlack ^a,b^, Markus Herderich ^b,e^, Kerry Wilkinson ^a,b*^

^a^ Discipline of Wine Science, Adelaide University, PMB 1, Glen Osmond, SA 5064, Australia

^b^ Waite Research Institute, Adelaide University, PMB 1, Glen Osmond, SA 5064, Australia

^c^ VAF Memstar, PO Box 794, Nuriootpa, SA 5355, Australia

^d^ School of Chemical Engineering, Adelaide University, North Tce, Adelaide, SA 5005, Australia

^e^ The Australian Wine Research Institute, PO Box 46, Glenside, SA, 5065, Australia

* Corresponding Author: Professor Kerry Wilkinson, telephone: + 61 8 8313 7360, facsimile: + 61 8 8313 7716, email: [kerry.wilkinson@adelaide.edu.au](about:blank)

|  | **Table of Contents** | **Page** |
| --- | --- | --- |
| **Figure S1** | The schematic of solid phase adsorption coupled Micro AA bench top filtration unit, equipped with one nanofiltration membrane (MWCO < 1000 Da). | **S3** |
| **Figure S2** | Concentration of volatile phenols (µg/L) in column effluent (and cumulative percentage removal in total eluate) during preliminary resin regeneration trial, involving elution with 2% aqueous sodium hydroxide, 2% aqueous citric acid, and model wine. | **S4** |
| **Figure S3** | Concentration of phenolic glycosides (µg/L) in column effluent (and cumulative percentage removal in total eluate) during preliminary resin regeneration trial, involving elution with 2% aqueous sodium hydroxide, 2% aqueous citric acid, and model wine. | **S5** |
| **Figure S4** | Concentration of volatile phenols (µg/L) in column effluent (and cumulative percentage removal relative to total elution achieved by 2% aqueous sodium hydroxide) during resin regeneration trials, comparing elution with (a) 2% aqueous sodium hydroxide vs 5% aqueous sodium carbonate, pH adjusted to (b) 8, (c) 10 and (d) 12, followed by 2% aqueous citric acid, and model wine. | **S6** |
| **Figure S5** | Concentration of phenolic glycosides (µg/L) in column effluent (and cumulative percentage removal relative to total elution achieved by 20% aqueous isopropanol) during resin regeneration trials, comparing elution with (a) 6%, (b) 10% and (c) 20% aqueous isopropanol, followed by model wine. | **S7** |
| **Figure S6** | Concentration of phenolic glycosides (µg/L) in column effluent (and cumulative percentage removal relative to total elution achieved by 20% aqueous ethanol) during resin regeneration trials, comparing elution with (a) 6%, (b) 10% and (c) 20% aqueous ethanol, followed by model wine. | **S8** |
| **Figure S7** | Relative expansion of resin after soaking in 2% aqueous sodium hydroxide, water, and 20% or 50% aqueous isopropanol, for 48 h. | **S9** |
| **Figure S8** | Concentration of volatile phenols (µg/L) in column effluent (and cumulative percentage removal in total eluate) during resin regeneration trials, comparing elution with (a) 2% vs (b) 5% aqueous sodium hydroxide, followed by 2% citric acid in 20% aqueous ethanol. | **S10** |
| **Figure S9** | Concentration of phenol glycosides (µg/L) in column effluent (and cumulative percentage removal in total eluate) during resin regeneration trials, comparing elution with (a) 2% vs (b) 5% aqueous sodium hydroxide, followed by 2% citric acid in 20% aqueous ethanol. | **S11** |
| **Table S1** | Aroma and plate attributes used for sensory analysis of smoke tainted Chardonnay, rosé and Cabernet Sauvignon and their treated counterparts. | **S12** |
| **Table S2** | Concentration of volatile phenols (µg/L) in column effluent fractions (200 mL, 1 BV) collected during preliminary resin regeneration trial, i.e., eluting with 2% aqueous sodium hydroxide (fractions 1 to 5), 2% aqueous citric acid (fractions 6 to 10), and model wine (fractions 11 to 15). | **S13** |
| **Table S3** | Detection (response) of deuterium-labelled volatile phenol standards extracted from various basic and acidic eluents using pentane/ethyl acetate (2:1). | **S14** |
| **Table S4** | Detection (abundance) of syringol gentiobioside (SyrGG) and deuterium-labelled syringol gentiobioside (d_6_-SyrGG) from 2% aqueous NaOH after acidification and from model wine. | **S15** |
| **Table S5** | Total phenolic glycosides (µg/L) eluted by different aqueous alcoholic solutions (i.e., fractions 1 to 12), followed by model wine (i.e., fractions 13 to 15). | **S16** |
| **Table S6** | Concentration of volatile phenols (µg/L) in column effluent fractions (and cumulative percentage removal relative to 2% aqueous sodium hydroxide) during resin regeneration trial, i.e., eluting with 6%, 10% or 20% aqueous isopropanol (fractions 1 to 12), followed by model wine (fractions 13 to 15). | **S17** |
| **Table S7** | Concentration of volatile phenols (µg/L) in column effluent fractions (and cumulative percentage removal relative to 2% aqueous sodium hydroxide) during resin regeneration trial, i.e., eluting with 6%, 10% or 20% aqueous ethanol (fractions 1 to 12), followed by model wine (fractions 13 to 15). | **S18** |
| **Table S8** | Total volatile phenols (µg/L) eluted by different aqueous alcoholic solutions (i.e., in fractions 1 to 12) and following model wine (i.e., in fractions 13 to 15). | **S19** |
| **Table S9** | Concentration of volatile phenols (µg/L) in combined column effluent (i.e., fractions 1 to 3, 5, 7 and 9 to 15) collected during resin regeneration trial, i.e., eluting with 2% vs 5% aqueous sodium hydroxide (fractions 1 to 9), and then 2% citric acid in 20% aqueous ethanol (fractions 10 to 15). | **S20** |
| **Table S10** | Elution of total phenolic glycoconjugates (µg/L) by acidified 20% aqueous ethanol (fractions 10–15), following alkaline elution with either 2% or 5% aqueous NaOH. | **S21** |
| **Table S11** | Regeneration of the acidified 20% aqueous ethanol used for resin regeneration, by activated carbon. | **S22** |
| **Table S12** | Permeation and retention of free and glycosylated phenols (µg/L) during fractionation of smoke tainted Chardonnay, rosé and Cabernet Sauvignon wines using NF, and their effluent concentrations post adsorbent treatments. | **S23** |
| **Table S13** | Mean intensity rating for sensory attributes of smoke tainted Chardonnay, rosé and Cabernet Sauvignon wines before and after remediation via a combined NF and solid-phase adsorption treatment (using either a fixed-bed column packed with resin or an activated carbon cartridge). | **S24** |
| **Table S14** | Basic composition of smoke tainted Chardonnay, rosé and Cabernet Sauvignon wines before and after remediation via a combined NF and solid-phase adsorption treatment (using either a fixed-bed column packed with resin or an activated carbon cartridge). | **S25** |

**Fig. S1.** The schematic of solid phase adsorption (column-paced resin and activated carbon cartridge, bed volume = 200 and 300 mL respectively) coupled Micro AA bench top filtration unit, equipped with one nanofiltration membrane (MWCO < 1000 Da).

*
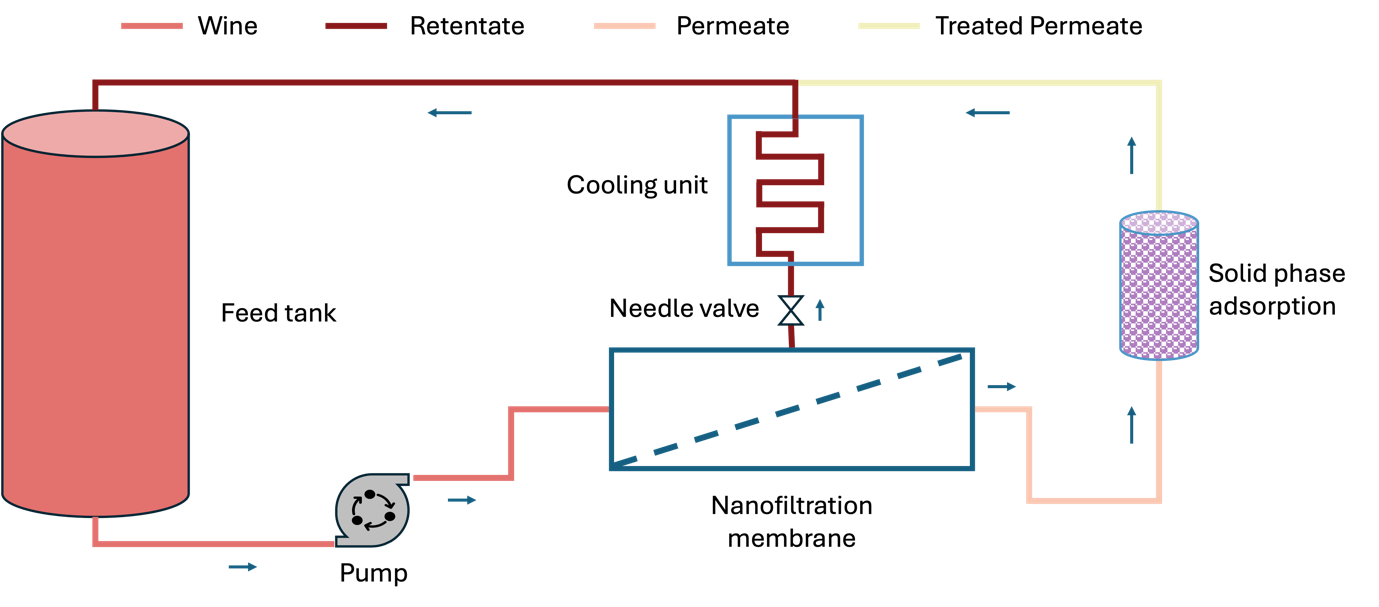
*

**Fig. S2.** Concentration of volatile phenols (µg/L) in column effluent (and cumulative percentage removal in total eluate, ) during preliminary resin regeneration trial, involving elution with 2% aqueous sodium hydroxide (fractions 1 to 5, blue background), 2% aqueous citric acid (fractions 6 to 10, pink background), and model wine (fractions 11 to 15, green background).


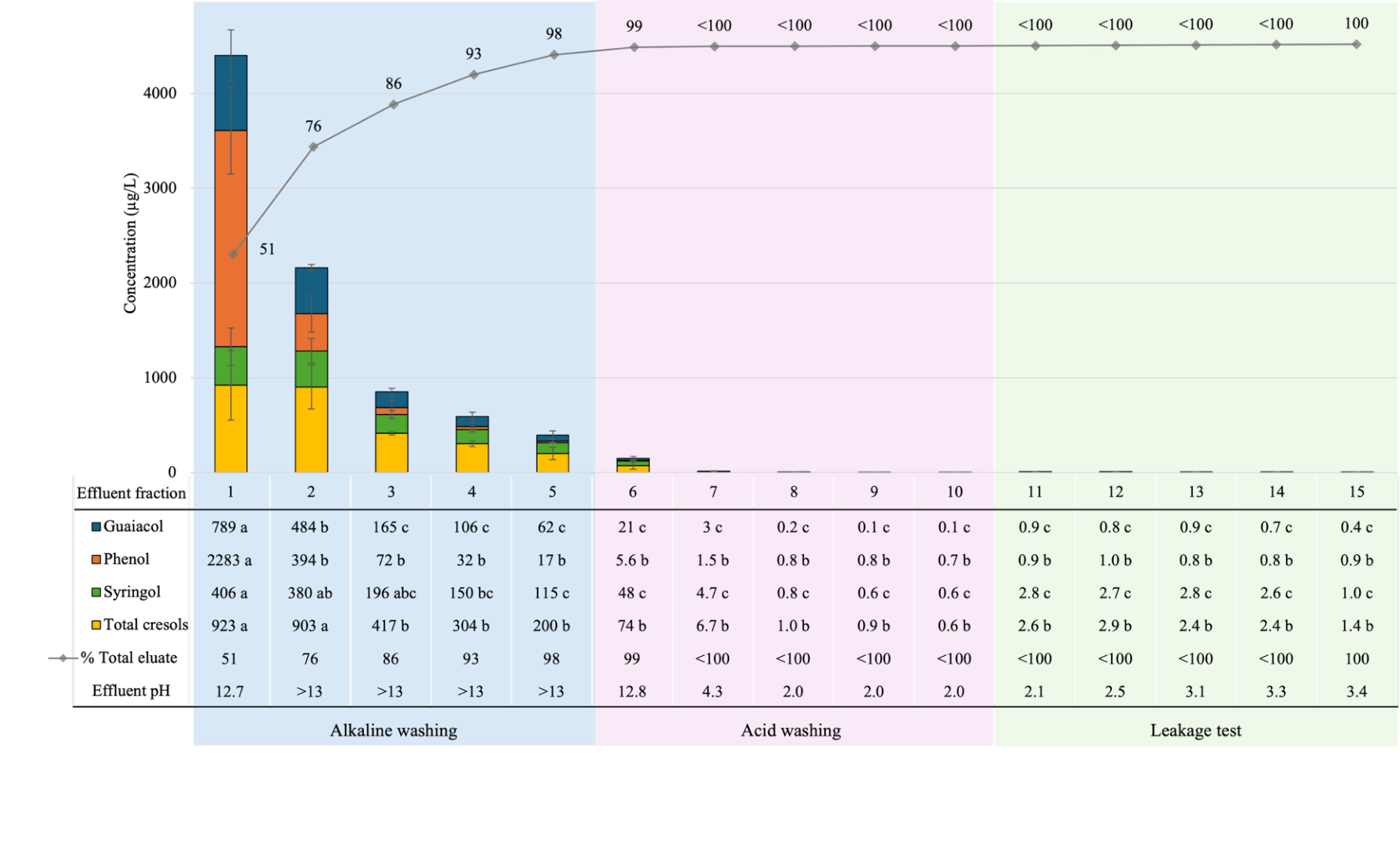


Data are means of two replicates (n = 2), with error bars representing standard deviation. Different letters (within rows) indicate statistical significance (P ≤0.05, one-way ANOVA). Model wine = 12% aqueous ethanol saturated with KHT and pH adjusted to 3.4. The resin bed volume and the volume of each effluent fraction were 200 mL.

**Fig. S3.** Concentration of phenolic glycosides (µg/L) in column effluent (and cumulative percentage removal in total eluate, ) during preliminary resin regeneration trial, involving elution with 2% aqueous sodium hydroxide (fractions 1 to 5, blue background), 2% aqueous citric acid (fractions 6 to 10, pink background), and model wine (fractions 11 to 15, green background).


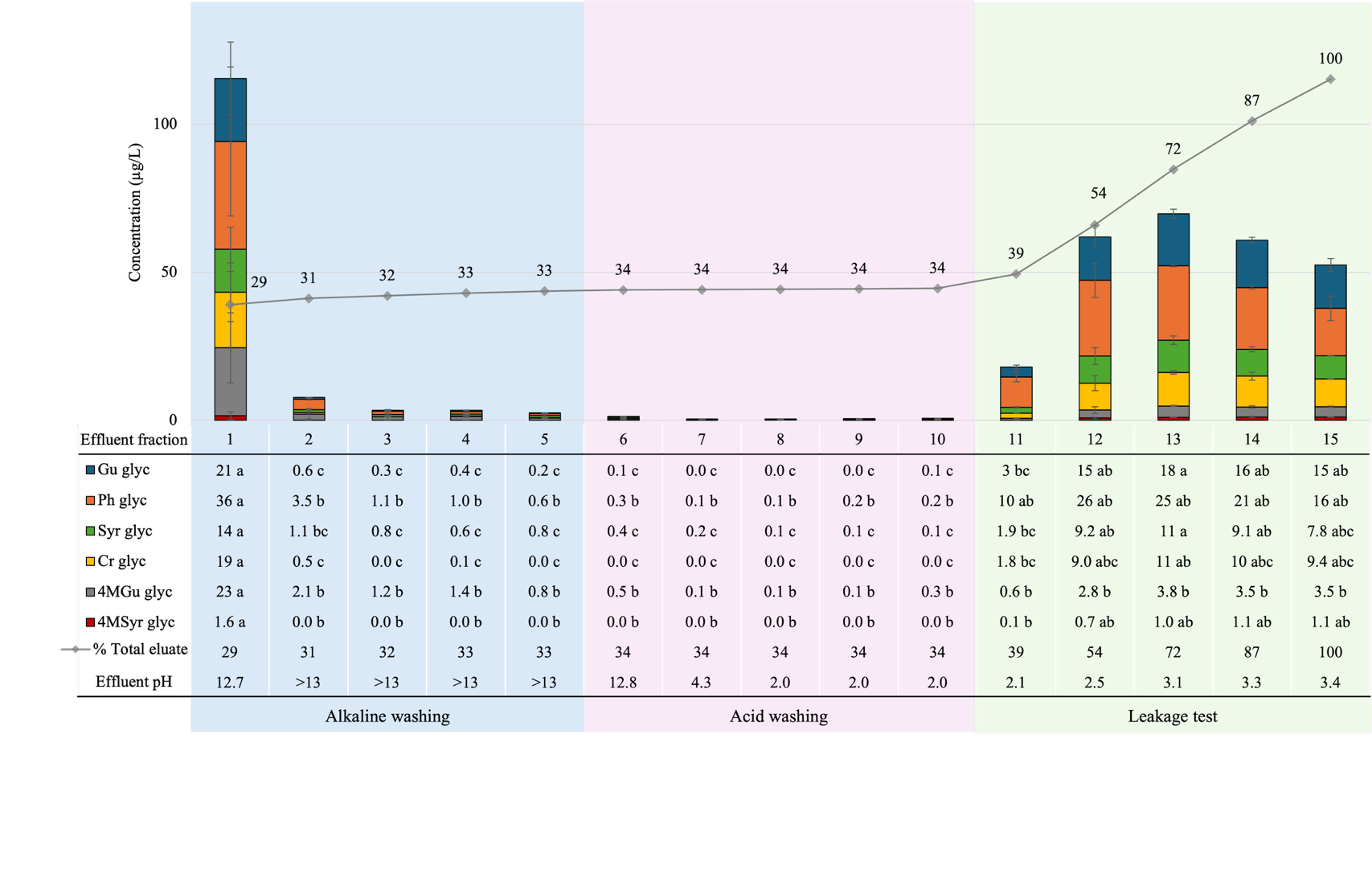


Data are means of two replicates (n = 2), with error bars representing standard deviation. Different letters (within rows) indicate statistical significance (P ≤0.05, one-way ANOVA). Model wine = 12% aqueous ethanol saturated with KHT and pH adjusted to 3.4. The resin bed volume and the volume of each effluent fraction were 200 mL. Glyc = glycosides, Gu = guaiacol, 4MGu = 4-methylguaiacol, Cr = cresol, Ph = phenol, Syr = syringol, 4MSyr = 4-methylsyringol. Phenolic glycosides were measured as syringol gentiobioside equivalents.

**Fig. S4.** Concentration of volatile phenols (µg/L) in column effluent (and cumulative percentage removal relative to total elution achieved by 2% aqueous sodium hydroxide, ) during resin regeneration trials, comparing elution with (a) 2% aqueous sodium hydroxide vs 5% aqueous sodium carbonate, pH adjusted to (b) 8, (c) 10 and (d) 12 (fractions 1 to 5, blue background), followed by 2% aqueous citric acid (fractions 10 to 12, pink background), and model wine (fractions 13 to 15, green background).


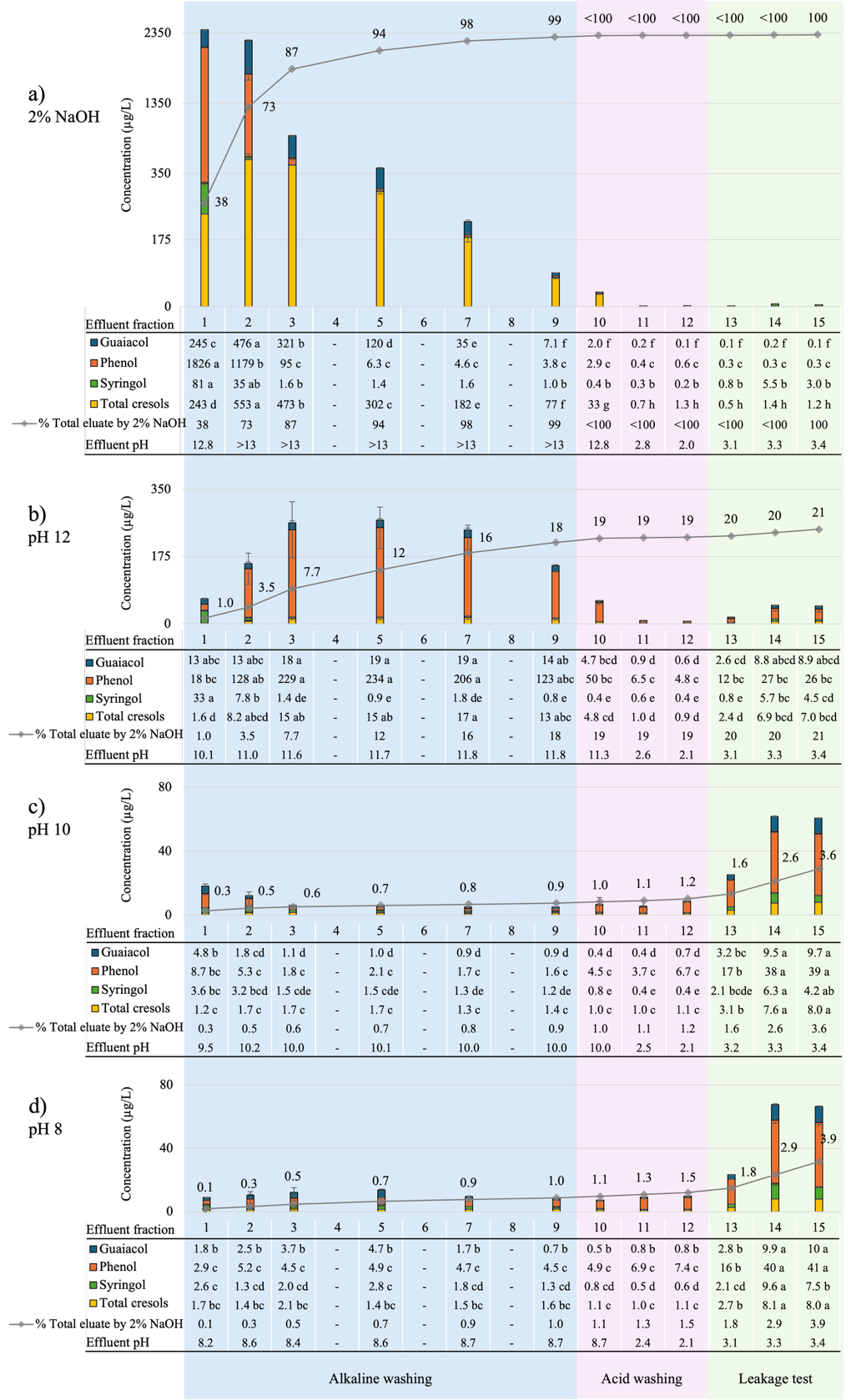


Data are means of two replicates (n = 2), with error bars representing standard deviation. Different letters (within rows) indicate statistical significance (P ≤0.05, one-way ANOVA). Model wine = 12% aqueous ethanol saturated with KHT and pH adjusted to 3.4. The resin bed volume and the volume of each effluent fraction were 50 mL.

**Fig. S5.** Concentration of phenolic glycosides (µg/L) in column effluent (and cumulative percentage removal relative to total elution achieved by 20% aqueous isopropanol (ISO), ) during resin regeneration trials, comparing elution with (a) 6%, (b) 10% and (c) 20% aqueous isopropanol (fractions 1 to 12, tan background), followed by model wine (fractions 13 to 15, green background).


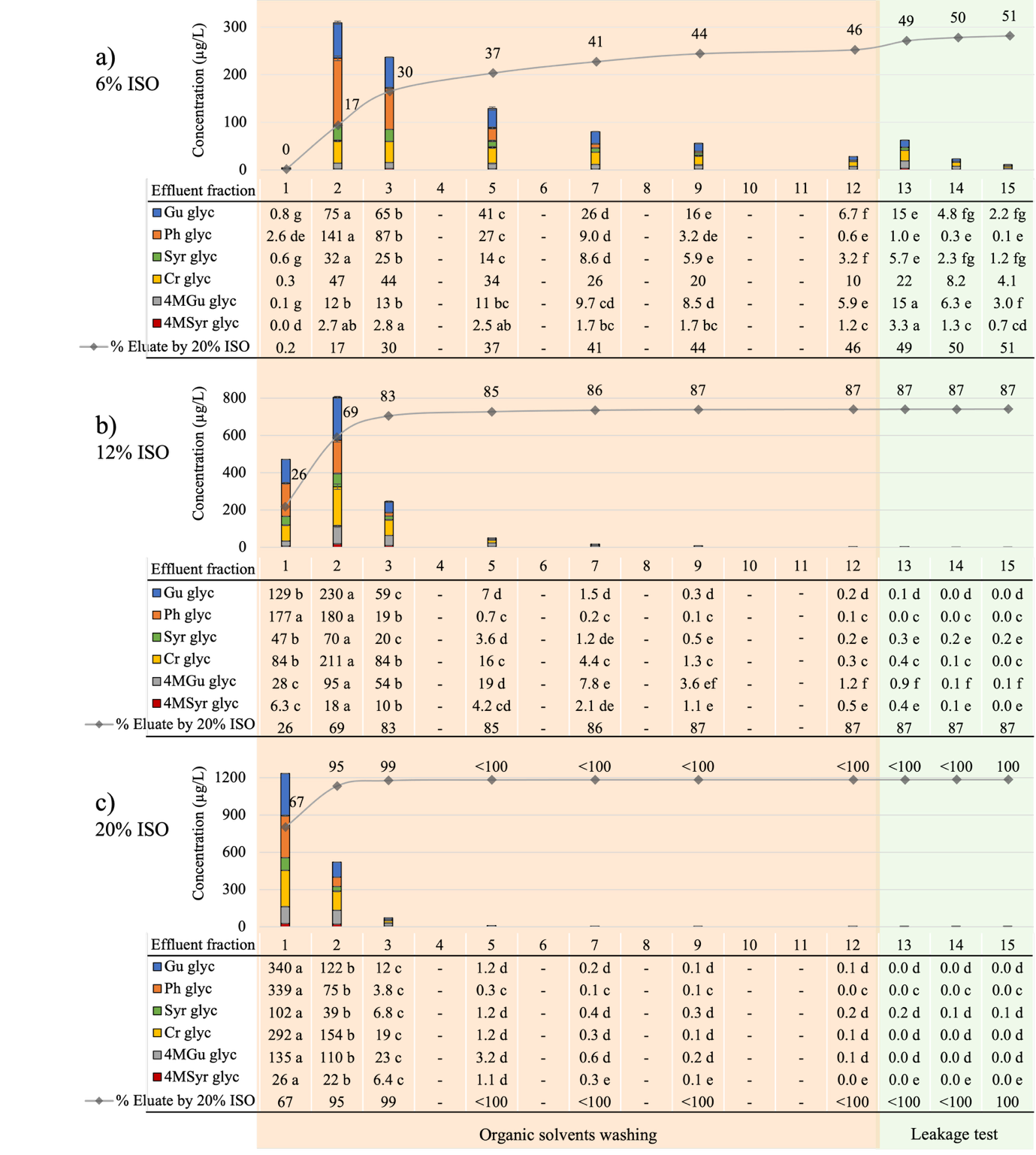


Data are means of two replicates (n = 2), with error bars representing standard deviation. Different letters (within rows) indicate statistical significance (*p* ≤0.05, one-way ANOVA). Model wine = 12% aqueous ethanol saturated with KHT and pH adjusted to 3.4. The resin bed volume and the volume of each effluent fraction were 50 mL. Glyc = glycosides, Gu = guaiacol, 4MGu = 4-methylguaiacol, Cr = cresol, Ph = phenol, Syr = syringol, 4MSyr = 4-methylsyringol. Phenol glycosides were measured as syringol gentiobioside equivalents.

**Fig. S6.** Concentration of phenolic glycosides (µg/L) in column effluent (and cumulative percentage removal relative to total elution achieved by 20% aqueous ethanol (EtOH), ) during resin regeneration trials, comparing elution with (a) 6%, (b) 10% and (c) 20% aqueous ethanol (fractions 1 to 12, tan background), followed by model wine (fractions 13 to 15, green background).


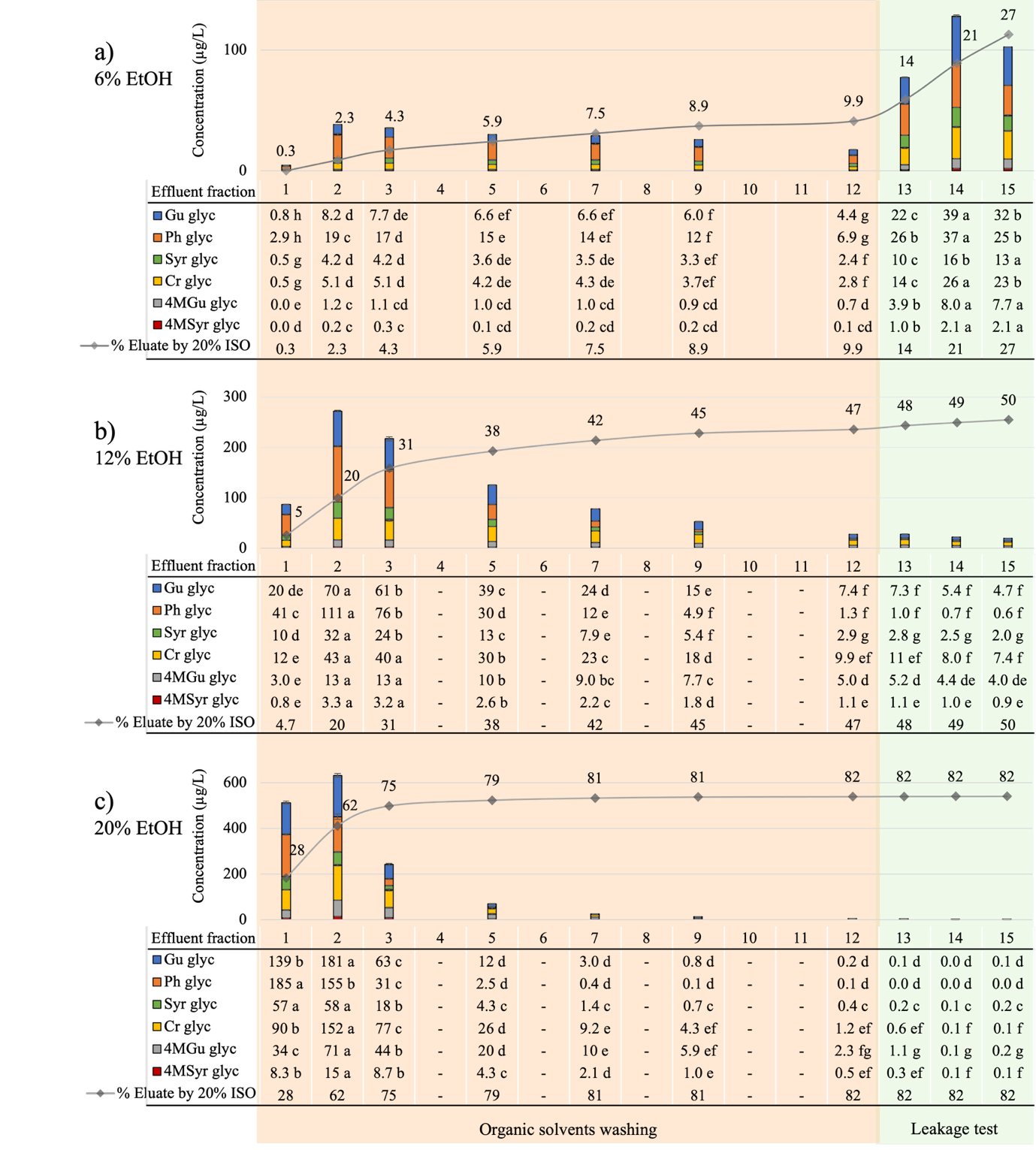


Data are means of two replicates (n = 2), with error bar representing standard deviation. Different letters (within rows) indicate statistical significance (p ≤0.05, one-way ANOVA). Model wine = 12% aqueous ethanol saturated with KHT and pH adjusted to 3.4. The resin bed volume and the volume of each effluent fraction were 50 mL. Glyc = glycosides, Gu = guaiacol, 4MGu = 4-methylguaiacol, Cr = cresol, Ph = phenol, Syr = syringol, 4MSyr = 4-methylsyringol. Phenol glycosides were measured as syringol gentiobioside equivalents.

**Fig. S7.** Relative expansion of resin after soaking in 2% aqueous sodium hydroxide (NaOH), water (H_2_O), and 20% or 50% aqueous isopropanol (ISO), for 48 h.


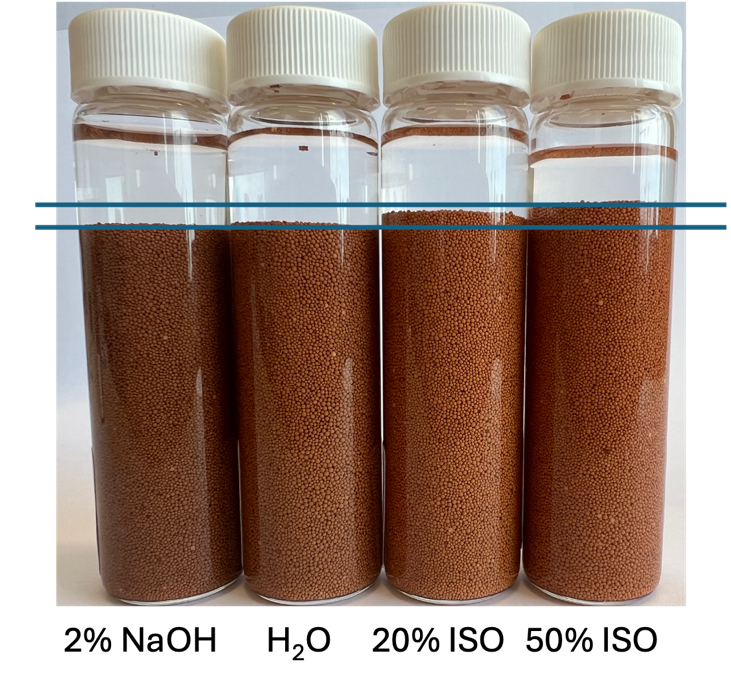


**Fig. S8.** Concentration of volatile phenols (µg/L) in column effluent (and cumulative percentage removal in total eluate, ) during resin regeneration trials, comparing elution with (a) 2% vs (b) 5% aqueous sodium hydroxide (fractions 1 to 9, blue background), followed by 2% citric acid in 20% aqueous ethanol (fractions 10 to 15, green background).


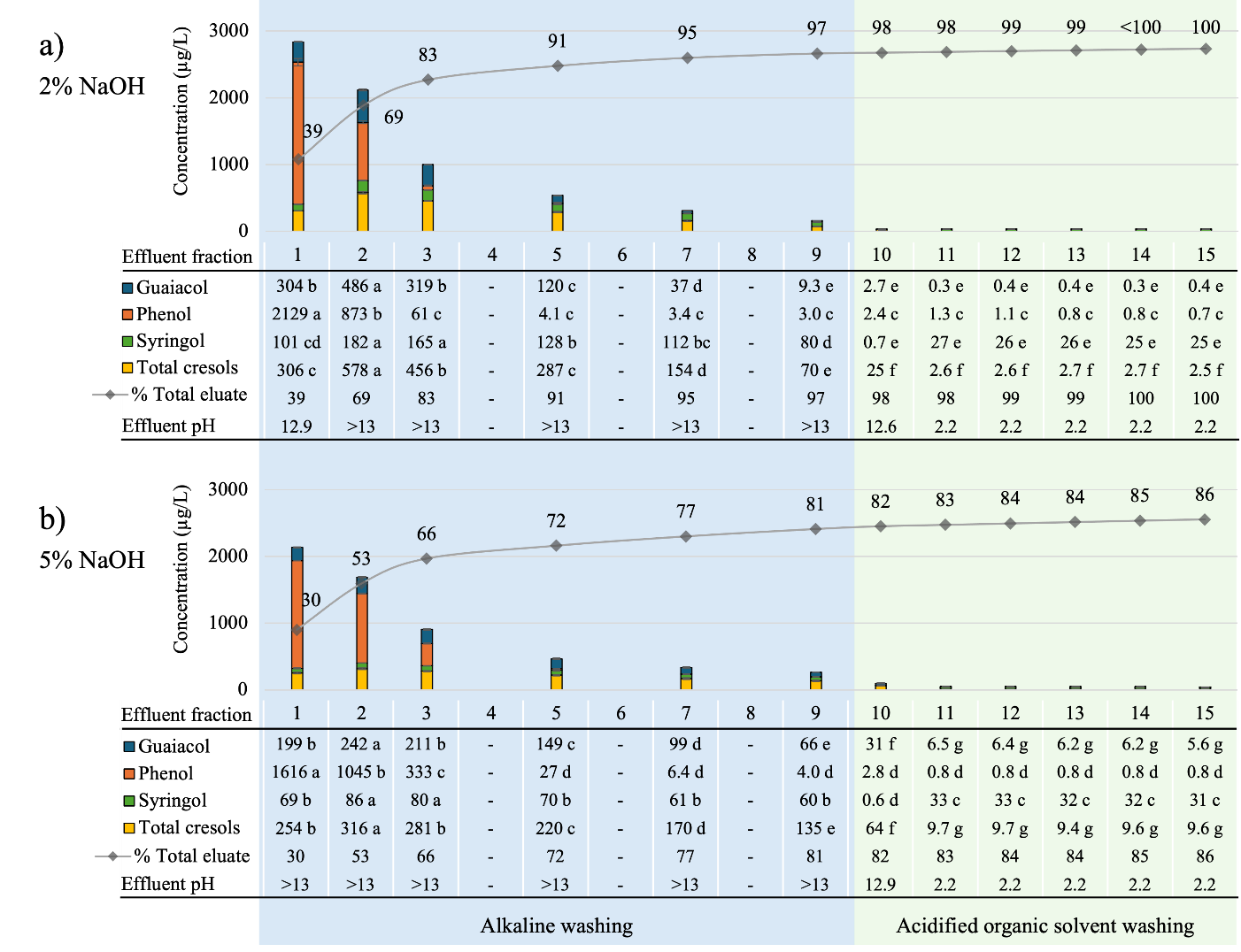


Data are means of two replicates (n = 2), with error bar representing standard deviation. Different letters (within rows) indicate statistical significance (P ≤0.05, one-way ANOVA). The resin bed volume and the volume of each effluent fraction were 50 mL.

**Fig. S9.** Concentration of phenol glycosides (µg/L) in column effluent (and cumulative percentage removal in total eluate, ) during resin regeneration trials, comparing elution with (a) 2% vs (b) 5% aqueous sodium hydroxide (fractions 1 to 9, blue background), followed by 2% citric acid in 20% aqueous ethanol (fractions 10 to 15, green background).


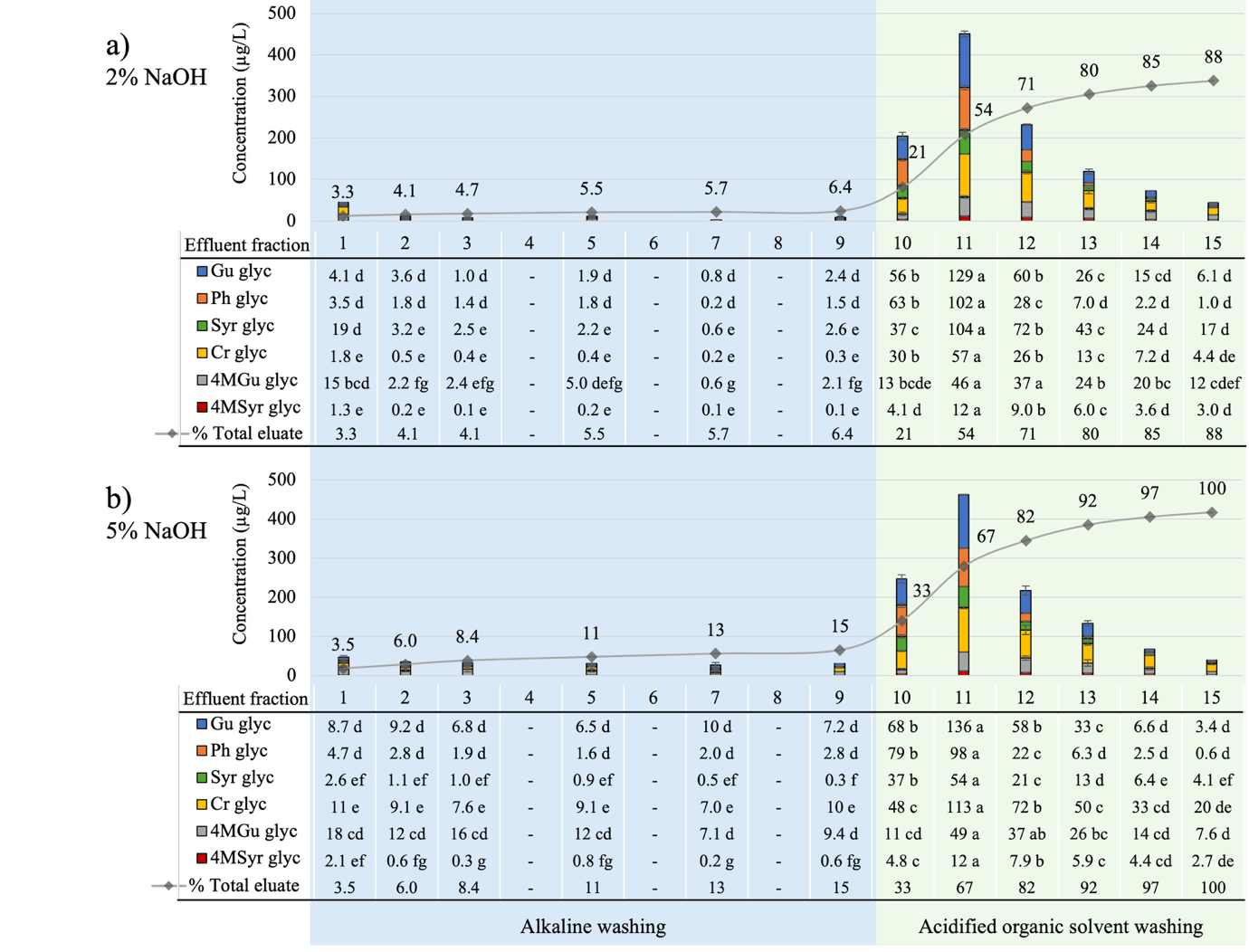


Data are means of two replicates (n = 2), with error bar representing standard deviation. Different letters (within rows) indicate statistical significance (P ≤0.05, one-way ANOVA). The resin bed volume and the volume of each effluent fraction were 50 mL. Glyc = glycosides, Gu = guaiacol, 4MGu = 4-methylguaiacol, Cr = cresol, Ph = phenol, Syr = syringol, 4MSyr = 4-methylsyringol. Phenol glycosides were measured as syringol gentiobioside equivalents.

**Table S1** Aroma and plate attributes used for sensory analysis of smoke tainted Chardonnay, rosé and Cabernet Sauvignon and their treated counterparts.

| Attributes | Description |
| --- | --- |
| *Aroma* |  |
| Overall fruit | Perception of aroma associated with fruit |
| Smoke | Perception of smoke aroma, including smoked meat/bacon, toasty, charry, cigar box |
| Cold ash | Perception of burnt aroma associated with ash, including ashtray, tarry, campfire |
| Earthy | Aromas associated with musty, dusty, wet wood, barnyard, mushroom, dank, moldy |
| Medicinal | Aromas characteristic of band-aids, disinfectant, cleaning products, solvents |
| Burnt rubber | Aromas associated with burnt rubber |
| Metallic | Perception of the ‘tinny’ aroma associated with metals |
| Oxidized | Perception of vinegar, bruised apple aroma, lost vibrancy |
| Reduced | Perception of stinky, rubber, sulfur, garlic |
| *Palate* |  |
| Acidity | Intensity of sour/acid taste |
| Bitterness | Intensity of bitter taste/aftertaste |
| Hotness | Intensity of hotness/warmth |
| Astringency | Intensity of perception of dry rough and puckery |
| Overall fruit | Perception of taste associated with fruit |
| Smoky | Perception of smoke flavour, including bacon and smoked meat |
| Medicinal | Perception of flavours characteristic of band-aids, disinfectant, cleaning products, solvents |
| Burnt rubber | Perception of flavours associated with burnt rubber |
| Metallic | Perception of the ‘tinny’ flavour associated with metals |
| Oxidized | Perception of vinegar, bruised apple flavours, lost vibrancy |
| Reduced | Perception of stinky, rubber, sulfur, garlic |
| *After taste* |  |
| Drying AT | Length of taste associated with drying, puckering mouthfeel after expectoration of the wine |
| Ashy AT | Length of taste associated with residue of ashtray perceived in the mouth after expectorating, including coal ash, ashtray, tarry, acrid, campfire |
| Woody AT | Length of taste associated with woody residue, including wood, oak, pencil shavings |

**Table S2** Concentration of volatile phenols (µg/L) in column effluent fractions (200 mL, 1 BV) collected during preliminary resin regeneration trial, i.e., eluting with 2% aqueous sodium hydroxide (fractions 1 to 5), 2% aqueous citric acid (fractions 6 to 10), and model wine (fractions 11 to 15).

| Fraction | Guaiacol | 4-Methyl guaiacol | *o*-Cresol | *m*-Cresol | *p*-Cresol | Phenol | Syringol | 4-Methyl syringol |
| --- | --- | --- | --- | --- | --- | --- | --- | --- |
| 1 | 789 a | 24 a | 209 ab | 375 a | 338 a | 2283 a | 406 a | 7.7 a |
| 2 | 484 b | 26 a | 224 a | 328 a | 352 a | 394 b | 380 ab | 6.0 ab |
| 3 | 165 c | 14 ab | 106 bc | 140 b | 171 ab | 72 b | 196 abc | 3.4 ab |
| 4 | 106 c | 13 ab | 79 c | 99 b | 126 b | 32 b | 150 bc | 2.7 ab |
| 5 | 62 c | 10 ab | 50 c | 60 b | 91 b | 17 b | 115 c | 2.5 ab |
| 6 | 21 c | 3.7 b | 18 c | 23 b | 34 b | 5.6 b | 48 c | 0.8 b |
| 7 | 3 c | 0.2 b | 1.4 c | 2.5 b | 2.9 b | 1.5 b | 4.7 c | 0.1 b |
| 8 | 0.2 c | 0.0 b | 0.2 c | 0.5 b | 0.3 b | 0.8 b | 0.8 c | 0.1 b |
| 9 | 0.1 c | 0.0 b | 0.2 c | 0.4 b | 0.3 b | 0.8 b | 0.6 c | 0.0 b |
| 10 | 0.1 c | 0.1 b | 0.1 c | 0.3 b | 0.2 b | 0.7 b | 0.6 c | 0.1 b |
| 11 | 0.9 c | 0.3 b | 0.5 c | 0.6 b | 1.5 b | 0.9 b | 2.8 c | 0.2 b |
| 12 | 0.8 c | 0.4 b | 0.5 c | 1.0 b | 1.4 b | 1.0 b | 2.7 c | 0.1 b |
| 13 | 0.9 c | 0.3 b | 0.5 c | 0.6 b | 1.3 b | 0.8 b | 2.8 c | 1.0 b |
| 14 | 0.7 c | 0.3 b | 0.5 c | 0.7 b | 1.3 b | 0.8 b | 2.6 c | 0.0 b |
| 15 | 0.4 c | 0.2 b | 0.3 c | 0.3 b | 0.7 b | 0.9 b | 1.0 c | 0.4 b |
| *P* | <0.0001 | 0.001 | <0.0001 | <0.0001 | <0.0001 | <0.0001 | <0.0001 | 0.007 |

Values are the mean of two replicates (n=2). Different letters (within columns) indicate statistical significance (*P* ≤0.05, one-way ANOVA). Model wine = 12% aqueous ethanol saturated with KHT and pH adjusted to 3.4.

**Table S3** Detection (response) of deuterium-labelled volatile phenol standards extracted from various basic and acidic eluents using pentane/ethyl acetate (2:1).

|  | *d*_4_-Guaiacol | *d*_5_-*o*-Cresol | *d*_6_-Phenol | *d*_3_-Syringol |
| --- | --- | --- | --- | --- |
| Acidification, post 2% aqueous  NaOH treatment overnight | 2.8 × 10^4^ a | 2.4 × 10^4^ a | 3.9 × 10^4^ a | 2.4 × 10^4^ a |
| Model wine | 2.7 × 10^4^ a | 2.5 × 10^4^ a | 3.9 × 10^4^ a | 2.2 × 10^4^ a |
| 2% aqueous citric acid | 2.9 × 10^4^ a | 2.4 × 10^4^ a | 3.8 × 10^4^ a | 2.4 × 10^4^ a |
| 2% aqueous NaOH (pH > 12) | 6.1 × 10^3^ b | 5.5 × 10^3^ b | 8.6 × 10^3^ b | 6.8 × 10^3^ b |
| *P* | <0.0001 | <0.0001 | <0.0001 | <0.0001 |

Values are means of three replicates (n=3). Different letters (within columns) indicate statistical significance (*P* ≤0.05, one-way ANOVA). Model wine = 12% aqueous ethanol saturated with KHT and pH adjusted to 3.4.

**Table S4** Detection (abundance) of syringol gentiobioside (SyrGG) and deuterium-labelled syringol gentiobioside (d_6_-SyrGG) from 2% aqueous NaOH after acidification and from model wine.

|  | SyrGG | *d*_6_-SyrGG |
| --- | --- | --- |
| Acidification, post 2% aqueous NaOH treatment overnight | 3.2 × 10^5^ | 8.7 × 10^6^ |
| Model wine | 3.2 × 10^5^ | 8.6 × 10^6^ |
| *P* | 0.706 | 0.555 |

Values are the mean of two replicates (n=2) ± standard deviation. Different letters (within columns) indicate statistical significance (*P* ≤0.05, *t*-test). Model wine = 12% aqueous ethanol saturated with KHT and pH adjusted to 3.4.

**Table S5** Total phenolic glycosides (µg/L) eluted by different aqueous alcoholic solutions (i.e., fractions 1 to 12), followed by model wine (i.e., fractions 13 to 15).

| Treatment | | Phenolic glycosides | |
| --- | --- | --- | --- |
|  |  | desorbed in  Fractions 1 to 12 | carried over in  Fractions 13 to 15 |
| Aqueous  ethanol | 6% | 181 ± 2 d | 308 ± 5 a |
|  | 12% | 859 ± 9 c | 69 ± 3 c |
|  | 20% | 1501 ± 65 b | 3.4 ± 0.2 d |
| Aqueous  isopropanol | 6% | 843 ± 28 c | 97 ± 3 b |
|  | 12% | 1599 ± 28 b | 3.0 ± 0.5 d |
|  | 20% | 1839 ± 3 a | 0.8 ± 0.1 d |
| *P* | | *<0.0001* | *<0.0001* |

Values are the mean of two replicates (n=2) ± standard deviation. Different letters (within columns) indicate statistical significance (*P* ≤0.05, one-way ANOVA). Model wine = 12% aqueous ethanol saturated with KHT and pH adjusted to 3.4. The resin bed volume and the volume of each effluent fraction were 50 mL.

**Table S6** Concentration of volatile phenols (µg/L) in column effluent fractions (and cumulative percentage removal relative to 2% aqueous sodium hydroxide) during resin regeneration trial, i.e., eluting with 6%, 10% or 20% aqueous isopropanol (fractions 1 to 12), followed by model wine (fractions 13 to 15).

| Solvent | Fraction | Gu | 4MGu | *o*-Cr | *m*-Cr | *p*-Cr | Ph | Syr | 4MSyr | Cumulative  removal (%) |
| --- | --- | --- | --- | --- | --- | --- | --- | --- | --- | --- |
| 6% aqueous isopropanol | 1 | 1.3 e | 0.1 e | 0.4 e | 0.2 d | 0.4 e | 6.7 f | 1.8 e | 0.9 | 0.2 |
|  | 2 | 10 cd | 0.6 abc | 1.9 cd | 2.6 bc | 3.3 cd | 40 de | 11 cd | 1.0 | 1.3 |
|  | 3 | 11 c | 0.4 bcd | 2.1 cd | 2.9 b | 4.0 bc | 46 c | 14 c | 0.7 | 2.6 |
|  | 5 | 11 c | 0.4 cd | 2.0 cd | 3.0 b | 3.9 bc | 46 c | 13 cd | 0.5 | 3.8 |
|  | 7 | 11 c | 0.4 bcd | 2.0 cd | 2.9 bc | 4.0 bc | 46 c | 14 cd | 0.4 | 5.1 |
|  | 9 | 11 c | 0.5 bcd | 2.1 bcd | 2.8 bc | 3.9 bc | 45 cd | 14 bc | 1.6 | 6.4 |
|  | 12 | 8.7 d | 0.4 d | 1.6 d | 2.4 c | 3.1 d | 36 e | 11 d | 0.5 | 7.4 |
|  | 13 | 16 a | 0.7 a | 2.8 a | 4.1 a | 5.3 a | 59 a | 21 a | 0.8 | 9.1 |
|  | 14 | 14 b | 0.6 ab | 2.6 ab | 3.7 a | 4.9 a | 53 b | 17 b | 0.3 | 11 |
|  | 15 | 11 c | 0.5 bcd | 2.2 bc | 3.0 b | 4.2 b | 44 cd | 12 cd | 0.3 | 12 |
| *P* |  | *<0.0001* | *<0.0001* | *<0.0001* | *<0.0001* | *<0.0001* | *<0.0001* | *<0.0001* | *0.205* |  |
| 12% aqueous isopropanol | 1 | 11 de | 0.9 b | 2.2 c | 3.0 ef | 3.4 e | 28 f | 30 ab | 3.6 a | 1.3 |
|  | 2 | 27 a | 1.5 a | 4.2 a | 6.6 a | 8.3 a | 88 a | 41 a | 1.4 b | 4.1 |
|  | 3 | 27 a | 1.1 b | 4.2 a | 6.4 ab | 8.3 a | 90 a | 43 a | 1.3 b | 6.9 |
|  | 5 | 26 ab | 1.1 b | 4.1 a | 6.3 ab | 8.2 a | 85 ab | 40 a | 1.1 b | 9.6 |
|  | 7 | 25 ab | 0.9 b | 4.0 a | 6.2 ab | 7.8 ab | 83 ab | 38 a | 1.0 b | 12 |
|  | 9 | 24 b | 0.9 b | 3.9 a | 6.0 b | 7.5 b | 78 b | 37 a | 0.9 b | 15 |
|  | 12 | 18 c | 0.8 bc | 3.1 b | 4.5 d | 6.0 c | 60 d | 27 ab | 0.7 b | 17 |
|  | 13 | 20 c | 0.8 bc | 3.4 b | 5.0 c | 6.5 c | 68 c | 29 ab | 0.6 b | 19 |
|  | 14 | 12 d | 0.5 cd | 2.4 c | 3.4 e | 4.4 d | 46 e | 14 bc | 0.2 b | 20 |
|  | 15 | 9.7 e | 0.4 d | 2.0 c | 2.8 f | 3.8 de | 38 e | 10 c | 0.1 b | 21 |
| *P* |  | *<0.0001* | *<0.0001* | *<0.0001* | *<0.0001* | *<0.0001* | *<0.0001* | *0.000* | *0.004* |  |
| 20% aqueous isopropanol | 1 | 33 d | 4.4 a | 5.6 d | 7.3 e | 8.4 d | 65 e | 54 d | 7.1 a | 2.9 |
|  | 2 | 63 a | 3.5 b | 8.3 a | 13 a | 16 a | 175 a | 118 a | 3.8 b | 9.2 |
|  | 3 | 60 a | 3 c | 8.0 ab | 12 a | 16 a | 170 a | 111 a | 3.4 bc | 15 |
|  | 5 | 54 b | 2.7 d | 7.4 bc | 11 b | 14 b | 155 b | 98 b | 2.9 cd | 21 |
|  | 7 | 48 c | 2.4 e | 7.2 c | 11 bc | 14 b | 140 c | 87 c | 2.5 de | 26 |
|  | 9 | 46 c | 2.2 e | 7.0 c | 10 c | 13 b | 129 c | 80 c | 2.3 de | 30 |
|  | 12 | 33 d | 1.7 f | 5.7 d | 8.5 d | 11 c | 91 d | 57 d | 1.8 ef | 34 |
|  | 13 | 26 e | 1.2 g | 4.6 e | 6.7 e | 8.3 d | 77 e | 41 e | 0.9 fg | 36 |
|  | 14 | 11 f | 0.5 h | 2.4 f | 3.2 f | 4.0 e | 39 f | 13 f | 0.2 gh | 37 |
|  | 15 | 8.8 f | 0.4 h | 2.0 f | 2.6 f | 3.4 e | 32 f | 9.0 f | 0.1 h | 38 |
| *P* |  | *<0.0001* | *<0.0001* | *<0.0001* | *<0.0001* | *<0.0001* | *<0.0001* | *<0.0001* | *<0.0001* |  |

Values are the mean of two replicates (n=2). Different letters (within columns, by solvent) indicate statistical significance (*P* ≤0.05, one-way ANOVA). Model wine = 12% aqueous ethanol saturated with KHT and pH adjusted to 3.4. The resin bed volume and the volume of each effluent fraction were 50 mL. Gu = guaiacol, 4MGu = 4-methylguaiacol, Cr = cresol, Ph = phenol, Syr = syringol, 4MSyr = 4-methylsyringol.

**Table S7** Concentration of volatile phenols (µg/L) in column effluent fractions (and cumulative percentage removal relative to 2% aqueous sodium hydroxide) during resin regeneration trial, i.e., eluting with 6%, 10% or 20% aqueous ethanol (fractions 1 to 12), followed by model wine (fractions 13 to 15).

| Solvent | Fraction | Gu | 4MGu | *o*-Cr | *m*-Cr | *p*-Cr | Ph | Syr | 4MSyr | Cumulative  removal (%) |
| --- | --- | --- | --- | --- | --- | --- | --- | --- | --- | --- |
| 6% aqueous ethanol | 1 | 1.7 e | 0.1 c | 0.4 d | 0.1 e | 0.3 d | 8.3 e | 2.0 e | 0.7 a | 0.2 |
|  | 2 | 5.4 c | 0.2 bc | 1.2 c | 1.7 c | 2.1 c | 25 c | 5.0 cd | 0.4 ab | 0.9 |
|  | 3 | 5.4 c | 0.2 bc | 1.2 c | 1.7 c | 2.2 c | 25 c | 5.0 cd | 0.3 ab | 1.5 |
|  | 5 | 5.4 c | 0.2 bc | 1.1 c | 1.6 cd | 2.0 c | 26 c | 5.1 c | 0.2 b | 2.2 |
|  | 7 | 5.1 cd | 0.2 c | 1.1 c | 1.5 cd | 2.0 c | 26 c | 4.9 cd | 0.3 ab | 2.8 |
|  | 9 | 5.1 cd | 0.2 c | 1.1 c | 1.6 cd | 2.0 c | 25 c | 4.8 cd | 0.2 b | 3.5 |
|  | 12 | 4.5 d | 0.2 c | 1.0 c | 1.3 d | 1.7 c | 22 d | 4.0 d | 0.1 b | 4.0 |
|  | 13 | 7.8 b | 0.3 b | 1.5 b | 2.1 b | 2.7 b | 34 b | 8.2 b | 0.2 b | 4.9 |
|  | 14 | 12 a | 0.5 a | 2.1 a | 3.0 a | 3.8 a | 47 a | 13 a | 0.2 b | 6.2 |
|  | 15 | 11 a | 0.5 a | 2.1 a | 2.9 a | 3.8 a | 45 a | 12 a | 0.2 b | 7.4 |
| *P* |  | *<0.0001* | *<0.0001* | *<0.0001* | *<0.0001* | *<0.0001* | *<0.0001* | *<0.0001* | *0.012* |  |
| 12% aqueous ethanol | 1 | 6.6 f | 0.5 b | 1.2 d | 2.1 d | 2.4 c | 25 f | 12 c | 2.1 a | 0.9 |
|  | 2 | 15 a | 1.0 a | 2.6 a | 3.9 a | 4.8 a | 51 ab | 16 a | 0.6 b | 2.3 |
|  | 3 | 14 ab | 0.8 ab | 2.4 ab | 3.5 ab | 4.4 ab | 52 a | 16 a | 0.5 b | 3.8 |
|  | 5 | 13 abc | 0.6 b | 2.3 abc | 3.4 bc | 4.5 ab | 51 abc | 16 a | 0.4 b | 5.3 |
|  | 7 | 13 ab | 0.7 ab | 2.2 abc | 3.3 bc | 4.2 ab | 51 abc | 16 a | 0.5 b | 6.7 |
|  | 9 | 13 bcd | 0.6 b | 2.3 abc | 3.4 bc | 4.3 ab | 49 abcd | 15 ab | 0.4 b | 8.1 |
|  | 12 | 10 e | 0.5 b | 2.0 c | 2.9 c | 3.7 b | 38 e | 12 c | 0.3 b | 9.2 |
|  | 13 | 11 cde | 0.5 b | 2.1 bc | 3.1 bc | 4.1 ab | 45 bcde | 13 bc | 0.3 b | 10 |
|  | 14 | 11 de | 0.5 b | 2.1 bc | 3.0 c | 4.0 ab | 44 cde | 13 c | 0.2 b | 12 |
|  | 15 | 11 e | 0.4 b | 2.1 bc | 3.0 bc | 4.0 ab | 43 de | 12 c | 0.2 b | 13 |
| *P* |  | *<0.0001* | *0.001* | *<0.0001* | *<0.0001* | *<0.0001* | *<0.0001* | *<0.0001* | *0.012* |  |
| 20% aqueous ethanol | 1 | 17 f | 1.6 b | 2.9 d | 4.4 e | 5.0 e | 43 g | 36 d | 5.7 a | 1.9 |
|  | 2 | 34 a | 2.4 a | 5.3 a | 8.1 a | 10 a | 101 a | 48 a | 1.8 b | 5.2 |
|  | 3 | 30 bc | 1.6 b | 5.0 ab | 7.5 b | 9.4 b | 95 bc | 43 abc | 1.3 bc | 8.4 |
|  | 5 | 32 b | 1.7 b | 4.5 b | 6.8 c | 8.7 c | 100 ab | 47 ab | 1.5 bc | 11 |
|  | 7 | 28 c | 1.4 bc | 5.2 a | 7.6 b | 9.7 ab | 89 c | 40 bcd | 1.1 bcd | 14 |
|  | 9 | 26 d | 1.4 bc | 4.9 ab | 7.2 b | 9.1 bc | 83 d | 38 cd | 1.1 bcd | 17 |
|  | 12 | 20 e | 1.1 cd | 3.7 c | 5.7 d | 7.1 d | 63 e | 28 e | 0.8 cde | 19 |
|  | 13 | 15 f | 0.7 de | 3.0 d | 4.3 e | 5.3 e | 53 f | 20 f | 0.4 de | 21 |
|  | 14 | 9.4 g | 0.4 e | 1.9 e | 2.7 f | 3.4 f | 35 h | 10 g | 0.2 e | 22 |
|  | 15 | 8.9 g | 0.4 e | 1.9 e | 2.4 f | 3.2 f | 34 h | 9.1 g | 0.1 e | 23 |
| *P* |  | *<0.0001* | *<0.0001* | *<0.0001* | *<0.0001* | *<0.0001* | *<0.0001* | *<0.0001* | *<0.0001* |  |

Values are the mean of two replicates (n=2). Different letters (within columns, by solvent) indicate statistical significance (p ≤0.05, one-way ANOVA). Model wine = 12% aqueous ethanol saturated with KHT and pH adjusted to 3.4. The resin bed volume and the volume of each effluent fraction were 50 mL. Gu = guaiacol, 4MGu = 4-methylguaiacol, Cr = cresol, Ph = phenol, Syr = syringol, 4MSyr = 4-methylsyringol.

**Table S8** Total volatile phenols (µg/L) eluted by different aqueous alcoholic solutions (i.e., in fractions 1 to 12) and following model wine (i.e., in fractions 13 to 15).

| Treatment | | Volatile phenols | | |
| --- | --- | --- | --- | --- |
|  |  | desorbed in  fractions 1 to 12 | | carried over in  fractions 13 to 15 |
| 2% NaOH | | 6294 ± 91 a | 14 ± 0.2 d | |
| Ethanol | 6% | 254 ± 7 f | 217 ± 0.2 c | |
|  | 12% | 582 ± 9 e | 231 ± 7 c | |
|  | 20% | 1211 ± 9 c | 225 ± 3 c | |
| Isopropanol | 6% | 468 ± 8 e | 281 ± 3 b | |
|  | 12% | 1057 ± 26 d | 283 ± 3 ab | |
|  | 20% | 2128 ± 9 b | 298 ± 5 a | |
| p | | <0.0001 | <0.0001 | |

Values are the mean of two replicates (n=2) ± standard deviation. Different letters (within columns) indicate statistical significance (*p*≤0.05, one-way ANOVA). Model wine = 12% aqueous ethanol saturated with KHT and pH adjusted to 3.4. The resin bed volume and the volume of each effluent fraction were 50 mL.

**Table S9** Concentration of volatile phenols (µg/L) in combined column effluent (i.e., fractions 1 to 3, 5, 7 and 9 to 15) collected during resin regeneration trial, i.e., eluting with 2% vs 5% aqueous sodium hydroxide (fractions 1 to 9), and then 2% citric acid in 20% aqueous ethanol (fractions 10 to 15).

| Treatment | Guaiacol | 4-Methyl  guaiacol | *o*-Cresol | *m*-Cresol | *p*-Cresol | Phenol | Syringol | 4-Methyl  syringol |
| --- | --- | --- | --- | --- | --- | --- | --- | --- |
| 2% NaOH | 1279 ± 0.8 a | 56 ± 1.3 a | 453 ± 3.3 a | 675 ±3.9 a | 761 ± 3.3 a | 3080 ± 85 | 897 ± 29 a | 13 ± 0.3 a |
| 5% NaOH | 1028 ± 17 b | 34 ± 0.5 b | 385 ± 5.6 b | 519 ± 5.1 b | 583 ± 3.9 b | 3038 ± 9.0 | 586 ± 7.2 b | 9.2 ± 0.4 b |
| *P* | *0.002* | *0.002* | *0.005* | *0.001* | *0.000* | *0.561* | *0.005* | *0.010* |

Values are the mean of two replicates (n=2) ± standard deviation. Different letters (within columns) indicate statistical significance (*P* ≤0.05, one-way ANOVA). The resin bed volume and the volume of each effluent fraction were 50 mL.

**Table S10** Elution of total phenolic glycoconjugates (µg/L) by acidified 20% aqueous ethanol (fractions 10–15), following alkaline elution with either 2% or 5% aqueous NaOH.

|  | Fraction 10 | Fraction 11 | Fraction 12 | Fraction 13 | Fraction 14 | Fraction 15 |
| --- | --- | --- | --- | --- | --- | --- |
| Acidified 20% aqueous ethanol post 2% NaOH | 204 ± 23 | 451 ± 20 | 232 ± 7.6 | 134 ± 13 | 73 ± 7.7 | 44 ± 0.3 |
| Acidified 20% aqueous ethanol post 5% NaOH | 247 ± 25 | 462 ± 1.8 | 217 ± 31 | 119 ± 23 | 68 ± 4.3 | 39 ± 2.2 |
| *P* | 0.211 | 0.495 | 0.585 | 0.515 | 0.494 | 0.089 |

Values are the mean of two replicates (n=2) ± standard deviation. Statistical analysis indicated there were no significant differences (*P* ≤0.05, *t*-test).

**Table S11** Regeneration of the acidified 20% aqueous ethanol used for resin regeneration, by activated carbon.

|  | Total volatile  phenols  (μg/L) | Total phenolic  glycosides (μg/L) |
| --- | --- | --- |
| Mixed acidic 20% aqueous ethanol post **2%** NaOH (**pre**-activated carbon treatment) | 32 ± 0.3 b | 171 ± 23 a |
| Mixed acidic 20% aqueous ethanol post **2%** NaOH (**post**-activated carbon treatment) | 4.6 ± 0.1 c | 48 ± 5.2 b |
| Mixed acidic 20% aqueous ethanol post **5%** NaOH (**pre**-activated carbon treatment) | 49 ± 1.4 a | 186 ± 13 a |
| Mixed acidic 20% aqueous ethanol post **5%** NaOH (**post**-activated carbon treatment) | 4.1 ± 0.3 c | 50 ± 7.2 b |
| *P* | <0.0001 | 0.001 |

Values are the mean of two replicates (n=2) ± standard deviation. Different letters (within columns) indicate statistical significance (*P* ≤0.05, one-way ANOVA).

**Table S12** Permeation and retention of free and glycosylated phenols (µg/L) during fractionation of smoke tainted Chardonnay (Ch), rosé and Cabernet Sauvignon (CS) wines using NF, and their effluent concentrations post adsorbent treatments.

| Wine | Volume of  permeate treated (mL) | Gu | 4MGu | *o*-Cr | *m*-Cr | *p*-Cr | Ph | Syr | 4MSyr | Gu  glyc | 4MGu glyc | Ph  glyc | Cr  glyc | Syr  glyc | 4MSyr glyc | Total glyc |
| --- | --- | --- | --- | --- | --- | --- | --- | --- | --- | --- | --- | --- | --- | --- | --- | --- |
| Untreated Ch |  | 43 a | 11 a | 6.9 a | 18 a | 15 a | 61 a | 62 a | 18 a | 703 a | 268 a | 809 a | 610 a | 1067 a | 165 a | 3012 a |
| NF permeate |  | 34 b | 8.3 b | 5.1 b | 14 b | 11 b | 50 b | 54 b | 15 b | 93 b | 30 b | 169 b | 109 b | 47 b | 6.3 b | 344 b |
| NF retentate |  | 41 a | 10 a | 6.6 a | 17 a | 14 a | 59 a | 61 a | 18 a | 705 a | 272 a | 832 a | 634 a | 1086 a | 170 a | 3065 a |
| *P* |  | <0.0001 | <0.0001 | <0.0001 | <0.0001 | <0.0001 | <0.0001 | <0.0001 | <0.0001 | <0.0001 | <0.0001 | <0.0001 | <0.0001 | <0.0001 | <0.0001 | <0.0001 |
| Treated Ch permeate  (using fresh resin) | 200 | 0.0 | 0.0 | 0.0 | 0.0 | 0.1 | 0.4 | 0.1 | 0.1 | 0.5 | 0.1 | 2.6 | 0.2 | 0.8 | 0.0 | 4.2 |
|  | 1000 | 0.0 | 0.0 | 0.1 | 0.0 | 1.0 | 0.7 | 0.1 | 0.0 | 29 | 2.1 | 119 | 19 | 23 | 0.5 | 193 |
|  | 5000 | 0.0 | 0.0 | 0.1 | 0.0 | 1.0 | 0.6 | 0.2 | 0.1 | 58 | 7.0 | 135 | 54 | 30 | 1.7 | 285 |
| Treated Ch permeate  (using regenerated resin) | 200 | 0.8 | 0.1 | 0.1 | 0.1 | 0.5 | 0.3 | 0.2 | 0.0 | 7.2 | 2.7 | 23 | 10 | 6.6 | 0.4 | 50 |
|  | 1000 | 1.5 | 0.0 | 0.1 | 0.3 | 0.9 | 0.4 | 0.2 | 0.0 | 28 | 6.2 | 88 | 29 | 18 | 1.2 | 170 |
|  | 5000 | 1.7 | 0.0 | 0.2 | 0.4 | 1.0 | 0.5 | 0.3 | 0.0 | 56 | 13 | 124 | 64 | 27 | 2.6 | 287 |
| Treated Ch permeate  (using activated carbon) | 200 | 0.0 | 0.0 | 0.1 | 0.0 | 0.0 | 1.5 | 0.0 | 0.0 | 0.0 | 0.1 | 0.0 | 0.0 | 0.2 | 0.0 | 0.4 |
|  | 1000 | 0.0 | 0.0 | 0.1 | 0.0 | 0.0 | 1.2 | 0.1 | 0.0 | 0.1 | 0.1 | 0.0 | 0.0 | 0.1 | 0.0 | 0.3 |
|  | 5000 | 0.0 | 0.0 | 0.1 | 0.0 | 0.1 | 3.8 | 0.1 | 0.0 | 0.4 | 0.4 | 0.3 | 0.0 | 0.6 | 0.0 | 1.8 |
| Untreated rosé |  | 26 a | 2.9 a | 3.4 a | 9.0 a | 9.0 a | 39 a | 17 a | 3.2 a | 395 a | 79 a | 365 a | 237 a | 118 b | 13 a | 971 a |
| NF permeate |  | 22 b | 2.6 a | 2.7 b | 7.1 b | 6.9 b | 33 b | 16 a | 2.9 a | 54 b | 8.6 b | 81 b | 44 b | 5.7 c | 0.6 b | 150 b |
| NF retentate |  | 26 a | 2.9 a | 3.3 a | 8.7 a | 8.9 a | 39 a | 17 a | 3.1 a | 438 a | 85 a | 382 a | 250 a | 131 a | 15 a | 1051 a |
| *P* |  | 0.002 | 0.089 | 0.006 | 0.001 | 0.003 | 0.002 | 0.071 | 0.273 | 0.001 | 0.000 | 0.001 | 0.001 | <0.0001 | 0.000 | 0.000 |
| Treated rosé permeate  (using regenerated resin) | 200 | 0.3 | 0.0 | 0.2 | 0.2 | 0.3 | 0.3 | 0.4 | 0.1 | 2.2 | 1.1 | 4.5 | 4.1 | 1.3 | 0.2 | 13 |
|  | 1000 | 1.1 | 0.0 | 0.3 | 0.5 | 0.7 | 0.4 | 1.9 | 0.1 | 13 | 4.4 | 33 | 14 | 3.9 | 0.7 | 68 |
|  | 5000 | 1.1 | 0.0 | 0.3 | 0.5 | 0.8 | 0.4 | 2.7 | 0.1 | 28 | 5.0 | 50 | 24 | 3.9 | 0.7 | 111 |
| Untreated CS |  | 43 a | 5.7 a | 13 a | 17 a | 18 a | 88 a | 52 a | 4.1 a | 316 a | 86 a | 417 a | 191 a | 202 a | 26 a | 1046 a |
| NF permeate |  | 33 b | 4.4 b | 9.0 b | 13 b | 13 b | 70 b | 44 b | 3.5 b | 19 b | 3.7 b | 42 b | 16 b | 3.7 b | 0.4 b | 69 b |
| NF retentate |  | 43 a | 5.7 a | 13 a | 17 a | 19 a | 89 a | 53 a | 4.3 a | 314 a | 86 a | 425 a | 197 a | 197 a | 27 a | 1048 a |
| *P* |  | 0.002 | 0.003 | 0.005 | 0.005 | 0.008 | 0.004 | 0.004 | 0.010 | 0.001 | 0.001 | 0.001 | 0.000 | 0.001 | 0.001 | 0.001 |
| Treated CS permeate  (using regenerated resin) | 200 | 0.2 | 0.1 | 0.1 | 0.1 | 0.2 | 0.3 | 1.2 | 0.0 | 1.5 | 1.1 | 3.2 | 4.0 | 0.5 | 0.2 | 11 |
|  | 1000 | 0.9 | 0.2 | 0.2 | 0.4 | 0.7 | 0.4 | 3.5 | 0.1 | 5.7 | 3.8 | 17 | 9.4 | 1.9 | 0.6 | 39 |
|  | 5000 | 1.0 | 0.3 | 0.3 | 0.5 | 0.8 | 0.6 | 4.9 | 0.1 | 17 | 4.1 | 38 | 16 | 3.6 | 0.6 | 80 |

Data are means of two replicates (n = 2). Different letters (within columns, for each wine filtration fraction) indicate statistical significance (p ≤ 0.05, one-way ANOVA). Phenolic glycosides were measured as syringol gentiobioside equivalents. Gu = guaiacol; 4MGu = 4-methylguaiacol; Cr = cresol; Ph = phenol; Syr = syringol; 4MSyr = 4-methylsyringol; glyc = glycosides.

**Table S13** Mean intensity rating for sensory attributes of smoke tainted Chardonnay, rosé and Cabernet Sauvignon wines before and after remediation via a combined NF and solid-phase adsorption treatment (using either a fixed-bed column packed with resin or an activated carbon cartridge).

|  | Chardonnay | | | | *P* | rosé | | *P* | Cabernet Sauvignon | | *P* |
| --- | --- | --- | --- | --- | --- | --- | --- | --- | --- | --- | --- |
|  | Untreated | Treated  (fresh resin) | Treated  (regenerated resin) | Treated  (activated carbon) |  | Untreated | Treated  (regenerated resin) |  | Untreated | Treated  (regenerated resin) |  |
| Overall fruit A | 3.91 a | 3.20 bc | 3.26 b | 2.99 c | <0001 | 3.60 a | 3.23 b | 0.0067 | 3.82 | 3.69 | 0.2527 |
| Smoke A | 2.96 a | 2.52 b | 2.36 b | 2.37 b | 0.0122 | 2.31 a | 1.91 b | 0.0438 | 2.47 a | 2.03 b | 0.0247 |
| Cold ash A | 2.21 | 2.35 | 2.27 | 1.96 | 0.2495 | 1.87 | 1.62 | 0.1718 | 1.99 a | 1.50 b | 0.0076 |
| Earthy A | 1.79 | 1.62 | 1.63 | 1.97 | 0.2148 | 1.67 a | 1.26 b | 0.0047 | 1.99 | 2.08 | 0.5977 |
| Medicinal A | 1.22 | 1.54 | 1.44 | 1.43 | 0.3790 | 1.73 | 1.42 | 0.1341 | 1.64 | 1.40 | 0.1382 |
| Burnt rubber A | 1.10 ab | 1.44 a | 0.97 b | 1.20 ab | 0.0735 | 1.11 | 0.77 | 0.0541 | 1.06 | 0.96 | 0.5482 |
| Metallic A | 0.96 | 1.11 | 1.13 | 1.10 | 0.6352 | 1.15 | 1.18 | 0.8372 | 1.01 | 0.93 | 0.4512 |
| Oxidized A | 1.39 ab | 1.47 ab | 1.35 b | 1.78 a | 0.1748 | 1.39 | 1.46 | 0.6512 | 1.21 | 1.09 | 0.4063 |
| Reduced A | 0.88 b | 1.00 b | 0.91 b | 1.29 a | 0.0155 | 1.04 | 1.05 | 0.9426 | 0.80 | 0.64 | 0.1067 |
| Acidity | 4.19 | 4.00 | 3.93 | 3.88 | 0.0690 | 4.02 | 4.12 | 0.4068 | 3.62 | 3.48 | 0.0897 |
| Bitterness | 3.58 | 3.26 | 3.41 | 3.26 | 0.0720 | 3.11 | 2.80 | 0.0541 | 3.55 | 3.36 | 0.1155 |
| Hotness | 3.53 a | 3.17 b | 3.28 ab | 3.24 b | 0.0465 | 3.32 a | 3.11 b | 0.0245 | 3.40 | 3.41 | 0.9319 |
| Drying AT | 3.56 | 3.44 a | 3.37 | 3.44 | 0.4143 | 3.32 | 3.08 | 0.0537 | 4.27 | 4.44 | 0.1073 |
| Astringency | 3.43 a | 3.36 ab | 3.13 b | 3.22 ab | 0.0485 | 3.37 | 3.14 | 0.0820 | 4.39 | 4.30 | 0.4407 |
| Overall fruit F | 3.39 | 3.24 | 3.25 | 3.15 | 0.1174 | 3.49 | 3.32 | 0.1601 | 3.63 | 3.64 | 0.9241 |
| Smoky F | 3.02 a | 2.54 b | 2.42 b | 2.68 ab | 0.0111 | 2.29 a | 1.76 b | 0.0051 | 2.71 a | 2.13 b | 0.0012 |
| Ashy AT | 2.84 | 2.57 | 2.46 | 2.43 | 0.1649 | 2.23 a | 1.73 b | 0.0085 | 2.40 | 2.18 | 0.2237 |
| Woody AT | 1.90 | 1.73 | 1.91 | 1.92 | 0.6125 | 1.73 | 1.52 | 0.1154 | 2.38 | 2.60 | 0.2208 |
| Medicinal F | 1.38 | 1.52 | 1.21 | 1.32 | 0.3079 | 1.28 | 1.37 | 0.5409 | 1.40 | 1.25 | 0.2691 |
| Burnt rubber F | 1.06 ab | 1.26 a | 0.77 b | 0.98 ab | 0.0120 | 0.82 | 0.64 | 0.1567 | 1.11 | 0.86 | 0.2277 |
| Metallic F | 1.20 | 1.21 | 1.03 | 1.25 | 0.4656 | 1.31 | 1.19 | 0.4197 | 0.91 | 0.88 | 0.8006 |
| Oxidized F | 1.31 b | 1.54 b | 1.48 b | 1.97 a | 0.0009 | 1.27 | 1.47 | 0.1170 | 1.17 | 1.27 | 0.5223 |
| Reduced F | 0.73 b | 1.10 a | 0.76 b | 1.11 a | 0.0010 | 0.89 | 0.91 | 0.8523 | 0.89 | 0.80 | 0.4068 |

Values are means of rating from sensory panelists (n = 50). Different letters (within rows, by wine) indicate statistical significance (*P* ≤ 0.05, two-way ANOVA); A = aroma; F = flavor; AT = aftertaste.

**Table S14** Basic composition of smoke tainted Chardonnay, rosé and Cabernet Sauvignon wines before and after remediation via a combined NF and solid-phase adsorption treatment (using either a fixed-bed column packed with resin or an activated carbon cartridge).

|  | Chardonnay | | | | *P* | rosé | | *P* | Cabernet Sauvignon | | *P* |
| --- | --- | --- | --- | --- | --- | --- | --- | --- | --- | --- | --- |
|  | Untreated | Treated (fresh resin) | Treated  (regenerated resin) | Treated (activated carbon) |  | Untreated | Treated (regenerated resin) |  | Untreated | Treated  (regenerated resin) |  |
| pH | 3.48 ± 0.01 | 3.44 ± 0.00 | 3.48 ± 0.03 | 3.50 ± 0.01 | *ns* | 3.07 ± 0.00 | 3.08 ± 0.00 | *ns* | 3.77 ± 0.00 | 3.77 ± 0.00 | *ns* |
| TA (g/L) | 5.78 ± 0.04 b | 6.00 ± 0.04 a | 5.78 ± 0.04 b | 5.37 ± 0.05 c | *0.001* | 6.10 ± 0.06 | 6.08 ± 0.03 | *ns* | 4.95 ± 0.08 | 4.98 ± 0.01 | *ns* |
| Ethanol (g/L) | 109 ± 0.08 a | 105 ± 0.93 b | 108 ± 0.35 a | 107 ± 0.00 ab | *0.009* | 103 ± 0.02 | 102 ± 0.32 | *ns* | 117 ± 0.14 a | 116 ± 0.06 b | *0.017* |
| Total phenolics (au) | 0.85 ± 0.01 a | 0.73 ± 0.02 b | 0.75 ± 0.00 b | 0.65 ± 0.02 c | *0.001* | - | - | *-* | - | - | *-* |
| Browning (au) | 0.15 ± 0.00 b | 0.15 ± 0.00 b | 0.15 ± 0.00 b | 0.16 ± 0.00 a | *0.001* | - | - | *-* | - | - | *-* |
| Total anthocyanins (mg/L) | - | - | - | - | - | 9.28 ± 1.74 | 9.85 ± 2.20 | *ns* | 105 ± 7.93 | 108 ± 4.23 | *ns* |
| Color density (au) | - | - | - | - | - | 0.65 ± 0.12 | 0.61 ± 0.10 | *ns* | 10 ± 0.06 | 10 ± 0.07 | *ns* |
| Hue | - | - | - | - | - | 1.49 ± 0.05 | 1.70 ± 0.14 | *ns* | 0.86 ± 0.00 | 0.90 ± 0.00 | *ns* |
| Total phenolics (au) | - | - | - | - | - | 6.06 ± 0.17 | 5.27 ± 0.24 | *ns* | 45 ± 0.57 | 45 ± 0.88 | *ns* |
| Total red pigments (au) | - | - | - | - | - | 0.76 ± 0.13 | 0.79 ± 0.19 | *ns* | 12 ± 0.36 | 12 ± 0.18 | *ns* |

Values are the mean of two replicates (n=2) ± standard deviation. Different letters (within rows, by wine) indicate statistical significance (*P* ≤0.05, one-way ANOVA). TA = titratable acidity (as tartaric acid equivalents); ns = not significant.
